# HobPre: accurate prediction of human oral bioavailability for small molecules

**DOI:** 10.1186/s13321-021-00580-6

**Published:** 2022-01-06

**Authors:** Min Wei, Xudong Zhang, Xiaolin Pan, Bo Wang, Changge Ji, Yifei Qi, John Z. H. Zhang

**Affiliations:** 1 Shanghai Engineering Research Center of Molecular Therapeutics & New Drug Development, Shanghai Key Laboratory of Green Chemistry & Chemical Process, School of Chemistry and Molecular Engineering, East China Normal University, Shanghai, 200062 China; 2 NYU-ECNU Center for Computational Chemistry at NYU Shanghai, Shanghai, 200062 China; 3 Department of Medicinal Chemistry, School of Pharmacy, Fudan University, Shanghai, 201203 China; 4 Department of Chemistry, New York University, New York, NY 10003 USA; 5 Shenzhen Institute of Synthetic Biology, Shenzhen Institute of Advanced Technology, Chinese Academy of Sciences, Shenzhen, Guangdong China; 6 Collaborative Innovation Center of Extreme Optics, Shanxi University, Taiyuan, Shanxi 030006 China

**Keywords:** Classification, Oral bioavailability, ADMET, Molecular descriptors, Prediction

## Abstract

Human oral bioavailability (HOB) is a key factor in determining the fate of new drugs in clinical trials. HOB is conventionally measured using expensive and time-consuming experimental tests. The use of computational models to evaluate HOB before the synthesis of new drugs will be beneficial to the drug development process. In this study, a total of 1588 drug molecules with HOB data were collected from the literature for the development of a classifying model that uses the consensus predictions of five random forest models. The consensus model shows excellent prediction accuracies on two independent test sets with two cutoffs of 20% and 50% for classification of molecules. The analysis of the importance of the input variables allowed the identification of the main molecular descriptors that affect the HOB class value. The model is available as a web server at www.icdrug.com/ICDrug/ADMET for quick assessment of oral bioavailability for small molecules. The results from this study provide an accurate and easy-to-use tool for screening of drug candidates based on HOB, which may be used to reduce the risk of failure in late stage of drug development.

**Supplementary Information:**

The online version contains supplementary material available at 10.1186/s13321-021-00580-6.

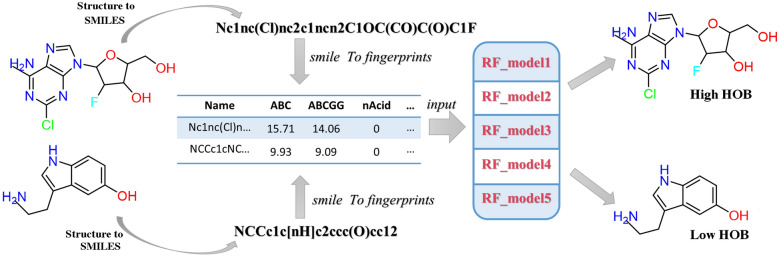

## Introduction

Poor pharmacokinetic properties, including absorption, distribution, metabolism, excretion, and toxicity (ADMET), are the key reasons of late-stage failures in drug development [1]. Therefore, ADMET assessments of candidate compounds during the early stages of drug discovery have become critical for improving the success rate of drug discovery and reducing the risk of late-stage attrition [2]. However, experimental testing of ADMET properties is time-consuming and costly. Thus, the accurate prediction of these properties is becoming increasingly important in drug discovery.

Among the ADMET properties, one of the most important pharmacokinetic characteristics of newly developed drugs is high oral bioavailability. Because oral administration is convenient and does not damage the skin or mucous membranes, 80% of the world’s drugs are administered orally [3]. Human oral bioavailability (HOB) is an important pharmacokinetic parameter that measures the amount of a drug that actually enters circulation within the body after ingestion. If intravenous administration is used, the human body can use the blood to deliver the drug to the site where it can exert pharmacological effects through the systemic circulation. Higher oral availability of the drug can reduce the amount of administration required to achieve the expected pharmacological effect because it can reduce the side effects and toxicity risks brought by the drug. On the other hand, poor oral bioavailability can lead to inefficiency of drugs and high inter-individual variability in the use of drugs, triggering some unpredictable drug reactions in the human body. In the actual drug development process, approximately 50% of candidate drugs fail due to low oral availability [4, 5]. Therefore, the level of oral availability is one of the key factors determining the success or failure of clinical trials of new drugs.

Experimental measurements of drug HOB are not only expensive, but also particularly time-consuming. Therefore, the development of a predictive model that can evaluate the HOB of a candidate compound before synthesis is of great help to drug discovery. Because the oral availability of a drug is affected by various biological, physical and chemical factors, such as the solubility of chemicals in the gastrointestinal tract, the permeability of the intestinal membrane, and the first pass metabolism of the intestine and liver, it is a very difficult and challenging task to develop accurate models to predict HOB. Nonetheless, a number of prediction models based on quantitative structure property relationships (QSPR) have been published [6, 7]. For example, Falcón-Cano et al. used 1448 molecules and obtained a consensus model with an accuracy of 78% [8]. Yang et al. used the random forest algorithm and 995 molecules to develop the admetSAR method with an accuracy of 69.7% [9]. On the basis of 995 data points, Kim et al. obtained 76% accuracy using the logistic classifier method [10].

In this study, we collected an HOB dataset composed of 1588 molecules and proposed a new model for HOB prediction based on machine learning. Using random forest (RF) [11–13] and two cutoffs of 20% and 50% for classifying molecules, we developed consensus models with a state-of-the-art accuracy on two independent test sets. Moreover, the importance of input molecular features to the prediction results was analyzed using the SHapley Additive exPlanation (SHAP) algorithm [14], revealing key molecular properties that affect HOB.

## Materials and methods

### Classification of positive and negative data

The performance of a classifier strongly depends on how the positive and negative samples are defined. However, there is still no consensus criterion to define positive and negative samples in HOB prediction. In previous studies, four cutoff values have been used: 20% (if HOB ≥ 20%, then the molecule belongs to the positive class; otherwise, it is a negative example) [15], 30% [16], 50% [17], and 80% [5] (Additional file 1: Table S1). We have therefor used two cutoffs 50% and 20% for labeling molecules in this study, which are used in more recent methods.

### Dataset preparation

The HOB training and test datasets from Falcón-Cano et al. [8] were used in this study, which includes 1157 training and 290 test molecules. The 290 test molecules (test set 1) were selected by randomly selecting 20% of all molecules. Three molecules in the test set 1 had wrong values and were manually corrected according to the relevant literatures [18–21]. An additional test set of 27 molecules was collected from a number of publications [8] and was then combined with the HOB data from ChEMBL to form an additional test set of 141 molecules (Test set 2, Table 1). To ensure that molecules in test set 2 do not overlap with the molecules in training set and test set 1, 2D structures were first generated and used to remove duplicates followed by deduplication using molecular fingerprints. Neither test set 1 nor test set 2 was used during the training.

**Table 1:** Information of the training and test datasets

Cutoff	Data sets	Molecules	Positive	Negative
F = 50%	Training set	1157	536	621
	Test set 1	290	169	121
	Test set 2	141	90	51
F = 20%	Training set	1142	859	283
	Test set 1	287	214	73
	Test set 2	133	128	5

The labeling of molecules using the 50% cutoff was obtained directly from Falcón-Cano et al. For the 20% cutoff, some molecules cannot be classified due to inaccurate experimental values. These molecules were discarded, leaving training set with 1142 molecules, test set 1 with 287 molecules and test set 2 with 133 molecules (Table 1).

All molecules in the training and test sets were converted to 3D structures using the RDKit package. However, the calculated 3D fingerprints and descriptors were not used during model training due to null value or small variance for many of the molecules.

**Fig. 1:**
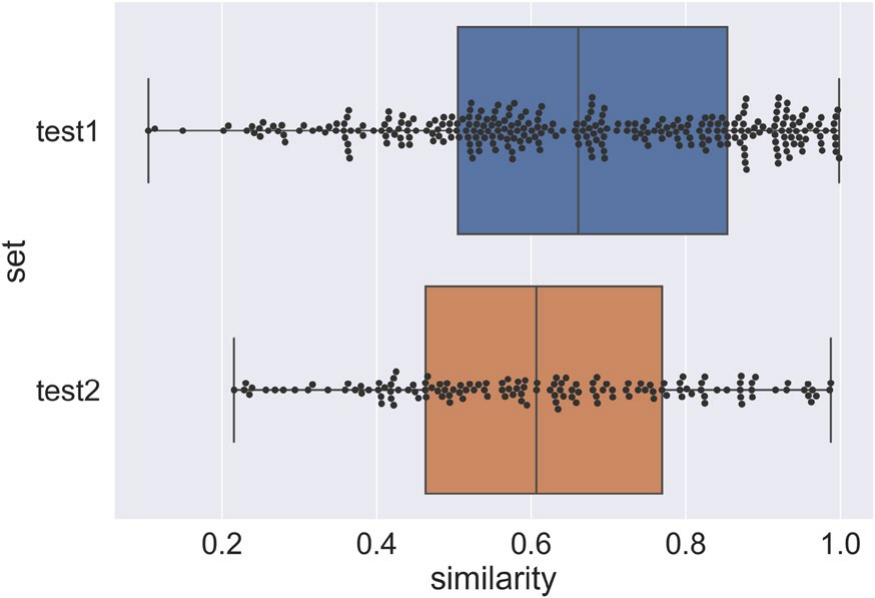
Maximum similarity of each molecule in the test sets to the molecules in the training set. The similarity was calculated using the Tanimoto coefficient similarity on the topological fingerprints of the molecules

To evaluate the similarity between the training set and the test sets, we calculated the max Tanimoto coefficient similarity of each molecule in the test sets with all molecules in the training set. The similarity ranges from 0.1 to 1 in test set 1 and 0.2 to 1 in test set 2 (Fig. 1). The average similarity between the test set 1 and the training set is 0.655, and the average similarity between the test set 2 and the training set is 0.612.

### Calculation of descriptors

We used Mordred [22] software to calculate 1614 molecular descriptors and fingerprints (Additional file 1: Table S2). Descriptors that had zero values or zero variance for all compounds were removed, reducing the total number of descriptors to 1143. All these 1143 features were used for training RF models.

### Evaluation of models

The performance of the individual and consensus models was evaluated by analyzing the sensitivity (SE), specificity (SP), accuracy of prediction (ACC), Matthew’s correlation coefficient (MCC) [23] and F1_score.

$$ACC=\frac{TP+TN}{TP+FP+TN+FN}$$

$$SE =\frac{TP}{TP+FN}$$

$$SP=\frac{TN}{TN+FP}$$

$$MCC=\frac{TP\times TN-FN\times FP}{\sqrt{(TP+FN)(TP+FN)(TN+FN)(TN+FP)}}$$

$$F{1}_{score}=2\times \frac{precision\times recall}{precision+recall}$$

where TP denotes true positive, FP is false positive, FN is false negative, and TN is true negative. In addition, the receiver operating characteristic (ROC) curve and the area under the curve (AUC) were also calculated.

We utilized the SHapley Additive exPlanation (SHAP) algorithm to explain the prediction model by providing consistent and locally accurate attribution values (SHAP values) for each feature within each prediction model [24]. The SHAP values evaluate the importance of the output resulting from the inclusion of a certain feature A for all combinations of features other than A.

## Results

### Performance of the models and comparison with previous methods

**Fig. 2:**
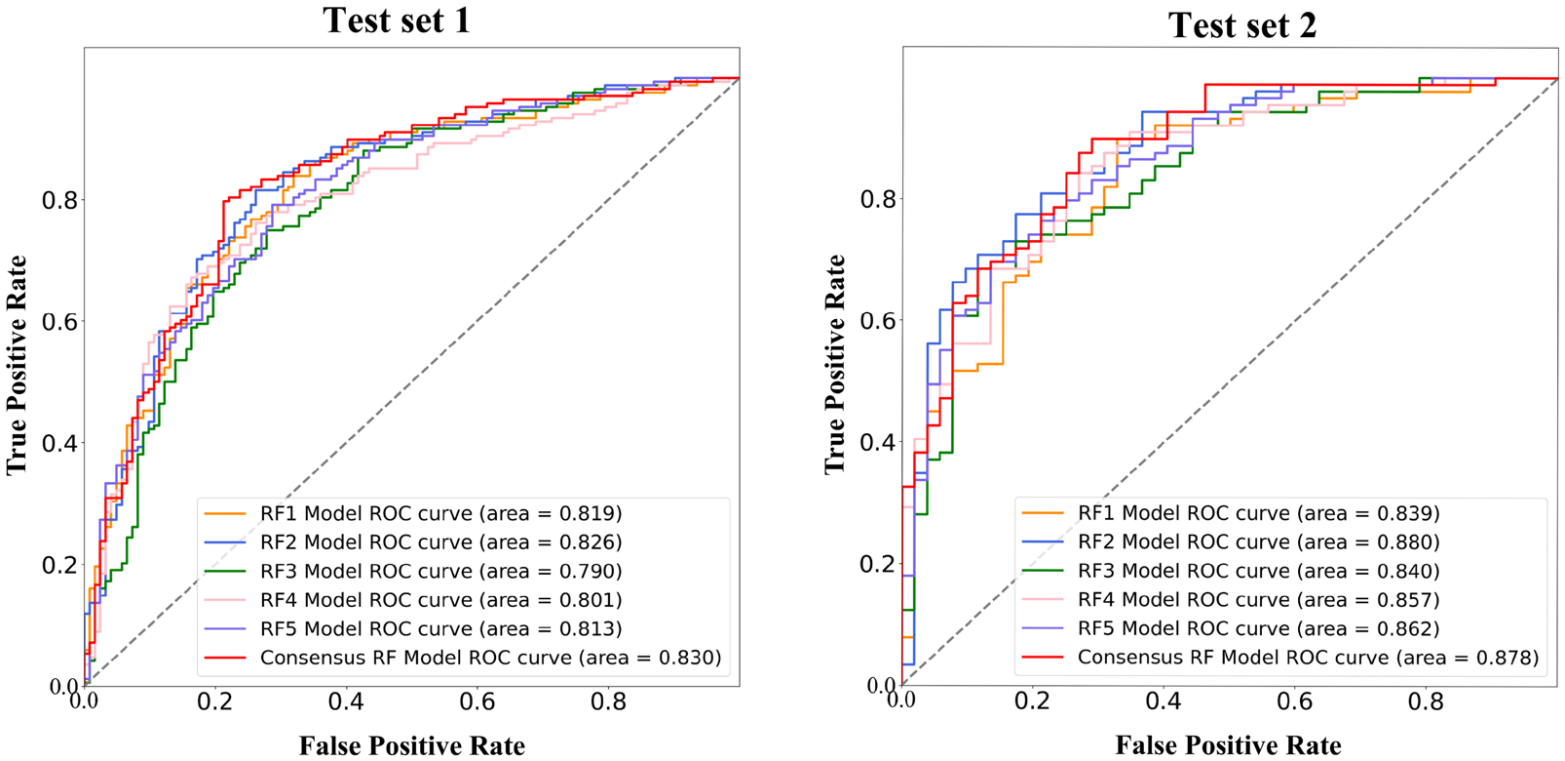
The AUCs of RF models on the two test sets when the cutoff is 50%

For the 50% cutoff, we first assembled a training set of 1157 molecules and two independent test sets with 290 and 146 molecules from the literature and public databases (see Materials and methods). The input features of the models include 1143 2D descriptors calculated with the Mordred package (see Materials and methods for details). Five individual RF models were first trained on the training set with fivefold cross-validation, using grid search and accuracy score (ACC) for hyperparameter optimization to obtain the best n_estimators and min_samples_leaf values while leaving the remaining hyperparameters set to default values (Table 2). Restricting the number of tunable hyper-parameters may reduce the risk of overfitting. The accuracy of the our model is 0.86–0.90 on the training set (Additional file 1: Tables S3, S4), and 0.74–0.77 on test set 1 (Table 3). The moderate decrease of accuracy on the test set suggests that our model has certain extend of overfitting but not severe. We then combined the five RF models to obtain a voting model. The final bioavailability class is the result of voting from each classification model with equal weight. Individual models can usually identify different aspects of the relationship between independent variables and dependent variables, and the relationships between the variables identified by those models may be different. In certain cases, the usage of a consensus model can greatly reduce the prediction error [25–27]. The accuracy of the five individual models ranges from 0.742 to 0.808 on the two test sets (Table 3). The consensus model shows improvement in accuracy on test set 1 and test set 2. The AUC values of the consensus model were 0.830 and 0.878 on the two test sets (Fig. 2). In addition, we merged the training set and test set 1, and used the same protocol to train five random forest models and obtained a consensus model from fivefold cross-validation. This whole process was repeated 50 times, and the average accuracy on test 2 was 0.826 with a standard deviation of 0.014, which was close to the accuracy of 0.823 when test set 1 is not included for training.

**Table 2:** Optimized parameters of the RF models from fivefold cross-validation on the training set when the cutoff is 50%

Parameters	Parameters meaning	Optimal value
n_estimators	The number of trees in the forest	31
min_samples_leaf	The minimum number of samples required to be at a leaf node	6

**Table 3:** Performance of the RF models on the two test sets when the cutoff is 50%

Data set	Model	SE	SP	ACC	AUC	MCC	F1-score
Test set 1	Model 1	0.779	0.732	0.752	0.819	0.505	0.774
	Model 2	0.746	0.786	0.769	0.826	0.529	0.798
	Model 3	0.713	0.750	0.734	0.790	0.460	0.766
	Model 4	0.745	0.732	0.738	0.801	0.473	0.764
	Model 5	0.713	0.768	0.745	0.813	0.479	0.777
	Consensus model	**0.787**	**0.797**	**0.793**	**0.830**	**0.580**	**0.817**
Test set 2	Model 1	0.673	0**.899**	0.816	0.839	0.596	0.860
	Model 2	0.673	0.876	0.801	**0.880**	0.565	0.848
	Model 3	0.577	0.854	0.752	0.840	0.452	0.813
	Model 4	**0.692**	0.865	0.801	0.857	0.568	0.846
	Model 5	0.673	0.831	0.773	0.862	0.509	0.822
	Consensus model	**0.692**	**0.899**	**0.823**	0.872	**0.612**	**0.865**

Bold numbers refer to the maximum value (optimum value) obtained from the corresponding evaluation index

To further estimate the accuracy of the models, the accuracy of our model on test set 1 was compared with those of previously published HOB models (Table 4). It should be noted that we used the same training and test sets as Falcón-Cano et al., but the accuracies of other models are directly taken from the respective literature publications, which may use different data sets.

**Table 4:** Comparison with other prediction models on test set 1 when the cutoff is 50%

Model	Data set size	Method	ACC (test)	AUC (test)	Cut-off value
Current study	1588	RF	0.793	0.830	F = 50%
Falcón-Cano et al. [8]	1448	CART, MLP, NB, GBT, SVM	0.783^a^	0.800^a^	F = 50%
admetSAR [9]	995	RF	0.697^a^	0.752^a^	logK(%F)^b^ = 0 (F = 50%)
Kim et al. [10]	995	RF, SVM-consensus CTG	0.76^a^	NA	F = 50%

*CART* classification and regression trees; *MLP* multilayer perceptron; *NB* naive Bayes; *GBT* gradient boosted trees; *SVM* support vector machines

^a^Taken from respective references

^b^$$\mathrm{log}K\left(\mathrm{\%}F\right)=\mathrm{log}\left(\frac{\mathrm{\%}F}{100-\mathrm{\%}F}\right)$$

Among these reported models, only admetSAR and ADMETlab provide an online prediction server that enables a direct comparison with our method based on the same test data. However, ADMETlab used different cutoffs of 30% and 20%. Therefore, we compared our method with admetSAR, which also uses F = 50% as the cut-off value. For a fair comparison, the molecules in the admetSAR training set were removed from the two test sets. Our model is more accurate than admetSAR in terms of SP, ACC and AUC (Table 5).

**Table 5:** Comparison with admetSAR on test sets 1 and 2 when the cutoff is 50%

Data set	Model	SE	SP	ACC	AUC
Test set 1^a^	Current Study	**0.824**	**0.819**	**0.821**	**0.862**
	admetSAR	0.784	0.777	0.780	0.831
Test set 2^a^	Current Study	0.692	**0.825**	**0.773**	**0.849**
	admetSAR	**0.769**	0.725	0.742	0.787

Bold numbers refer to the maximum value (optimum value) obtained from the corresponding evaluation index

^a^Test set 1 and 2 contain 168 and 66 molecules after removing the molecules used in the admetSAR training set

For the F = 20% cutoff, we used the same method to build a consensus model (Table 6). The accuracy of the five individual models ranges from 0.739 to 0.932 on the two test sets (Table 7). The consensus model shows improvement in accuracy on test set 1 and test set 2. The AUC values of the consensus model were 0.804 and 0.978 on the two test sets.

**Table 6:** Optimized parameters of the RF models from fivefold cross-validation on the training set when the cutoff is 20%

Parameters	Parameters meaning	Optimal value
n_estimators	The number of trees in the forest	10
min_samples_leaf	The minimum number of samples required to be at a leaf node	6

**Table 7:** Performance of the RF models on the two test sets when the cutoff is 20%

Data set	Model	SE	SP	ACC	AUC	MCC	F1-score
Test set 1 (287)	Model 1	0.370	0.869	0.742	0.736	0.264	0.824
	Model 2	0.479	0.864	0.767	0.767	0.360	0.847
	Model 3	0.452	0.893	0.780	0.771	0.379	0.858
	Model 4	0.452	0.856	0.746	0.759	0.308	0.832
	Model 5	0.493	0.855	0.763	0.789	0.359	0.833
	Consensus model	**0.493**	**0.925**	**0.815**	**0.801**	**0.473**	**0.882**
Test set 2 (133)	Model 1	0.8	0.891	0.887	0.973	0.384	0.938
	Model 2	1	0.859	0.865	0.978	0.432	0.924
	Model 3	0.8	0.883	0.880	0.939	0.371	0.933
	Model 4	1	**0.914**	0.917	0.955	0.534	**0.955**
	Model 5	1	0.898	0.902	0.973	0.499	0.946
	Consensus model	1	0.906	**0.910**	**0.981**	**0.516**	0.951

Bold numbers refer to the maximum value (optimum value) obtained from the corresponding evaluation index

The performance of our model is compared with that of ADMETlab [16] on the two test sets (Table 8). Our model showed lower ACC and AUC on test set 1 but better performance on test set 2. Because test set 1 comes from an earlier data set that was published before ADMETlab [8, 10, 28–30], and has overlapping molecules with the training sets of several previous methods, it is likely that some molecules in test set 1 may be included in the training of ADMETlab (We were not able to obtain the training set of ADMETlab for removing of redundancy). On the other hand, the HOB data in test set 2 does not overlap with any published training sets and may server as a more objective testing of the two models.

**Table 8:** Comparison with ADMETlab on test sets 1 and 2 when the cutoff is 20%

Data set	Model	SE	SP	ACC	AUC
Test set 1	Current study	0.493	**0.925**	0.815	0.801
	ADMETlab	**0.904**	0.855	**0.868**	**0.947**
Test set 2	Current study	**1**	**0.906**	**0.910**	**0.981**
	ADMETlab	0.8	0.844	0.842	0.902

Bold numbers refer to the maximum value (optimum value) obtained from the corresponding evaluation index

### Diversity distribution of HOB data

**Fig. 3:**
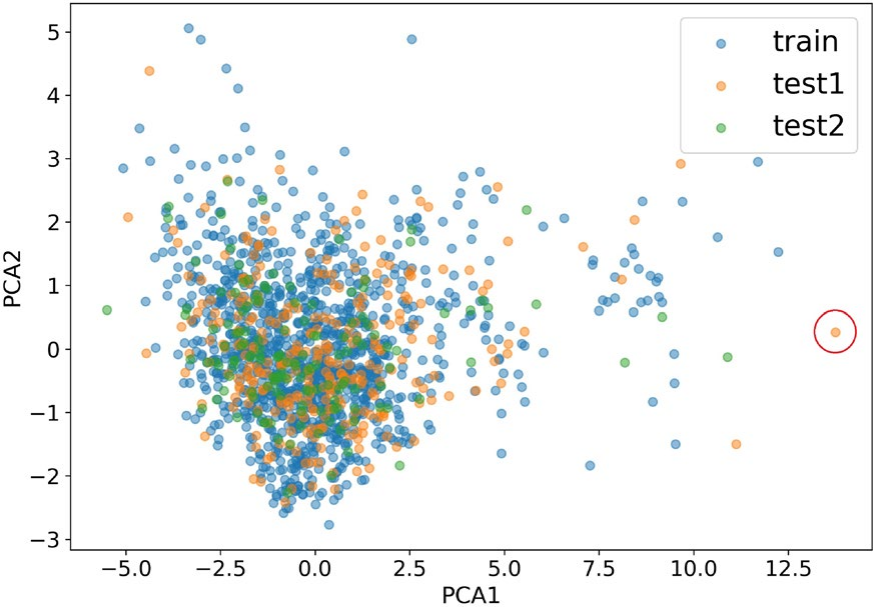
The chemical space of the training set (blue), the test set 1 (orange) and test set 2(green) using PCA factorization

In this study, 1157, 290 and 141 molecules that have human oral availability data were collected for model construction. To examine the diversity of these molecules and provide a possible way to access the applicability of our model, we carried out principal component analysis (PCA) of these molecules. The 1143 fingerprints and descriptors mentioned above for model training were used to generate PCA for all compounds. We selected the two most important components to create a chemical space for characterizing training set, test set 1, and test set 2 (Fig. 3). The results suggest that the chemical space of the two test sets is roughly within the space of the training set, therefore it is sensible to use the prediction model trained by the training set to predict the HOB values for the test sets. In addition, after removing an outlier in test set 1 (circled points in Fig. 3) that are outside the PCA space of the training set, the accuracy remains unchanged on test set 2 with 50% and 20% cutoff. Moreover, the PCA analysis can be used to determine the applicability of our model to new molecules. When the projection of a new molecule is within the range of the training molecules, it is considered as “inside” the application domain, indicating a more reliable prediction.

### Diversity evaluation of base learners

Diversity is very important in combining base learners. The Q-value approach is one way to measure the diversity of two classifiers [31]. It ranges between − 1 and 1, and is 0 if two classifiers are independent. The larger the Q value, the smaller the difference between the predictions of two classifiers. We used Q-value to measure the difference between the decision trees in each model. The average Q-value for individual trees in the five random forest models when the cutoff is 50% was 0.207, 0.233, 0.27, 0.241, and 0.267. When the cutoff is 20%, the Q-values were 0.235, 0.269, 0.288, 0.293, and 0.275, which suggest that these trees have high diversity.

### Importance of the input features

The analysis of important descriptors and fingerprints for prediction provides more information to fully understand these models. To this end, we used the SHapley Additive exPlanation (SHAP) algorithm to calculate the importance score of the input descriptor and fingerprints [14]. SHAP (SHapley Additive exPlanations) is a game theory method used to explain the output of a machine learning model. It uses the classical Shapley value from game theory and its related extension to link the optimal credit allocation with local explanation.

**Fig. 4:**
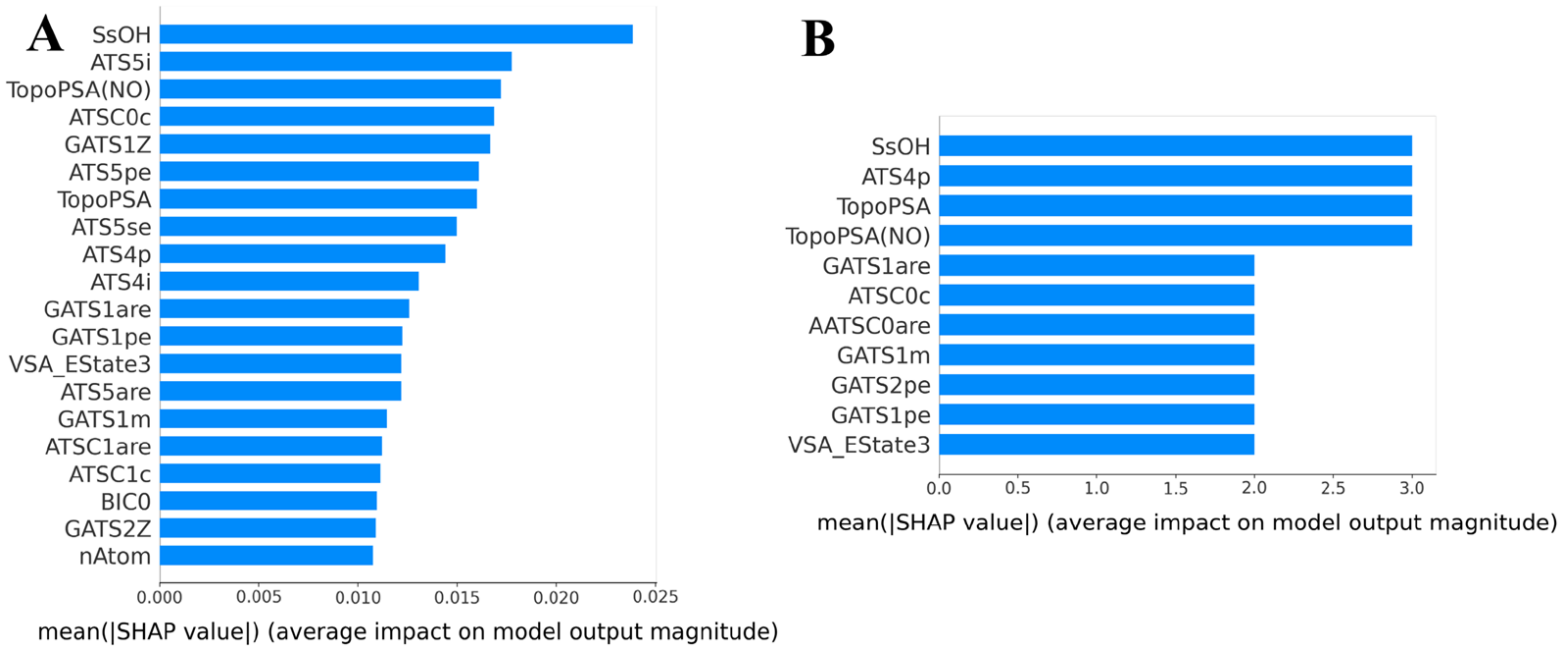
**A** Importance matrix plot of the consensus model when the cutoff is 50%. **B** A statistical graph of the number of occurrences of the top 20 features that affect all models when the cutoff is 50%

We used the same method to analyze the contribution of each descriptor in the RF models when the cutoff is 50%, and the top 20 most important variables that contributed to the model were obtained through importance matrix plots (Additional file 1: Figs. S1–S5). The importance matrix plot for the consensus method was calculated by averaging the value in each model (Fig. 4A), which depicts the importance of each input feature in the development of the final predictive model. SsOH (number of all atoms) which is an atom type e-state descriptor contributes the most to predictive power, followed by the topological structure descriptor ATS5i and polar surface area descriptor TopoPSA (NO). In addition, we also counted the number of appearances of the features in the five individual models (Fig. 4B). SsOH, ATS4p and TopoPSA(NO) appear three times among the 20 most important descriptors of the five models. In the two methods of quantifying feature importance, the specific information of the top 20 features is sorted in Table 9.

**Table 9:** Description of the important features in the consensus model when the cutoff is 50%

Descriptor category	Feature name	|shap value|	Description
Estate	SsOH	0.0239	Sum of sOH
TopoPSA	TopoPSA(NO)	0.0178	Topological polar surface area (use only nitrogen and oxygen)
Autocorrelation	ATSC0c	0.0172	Centered Moreau–Broto autocorrelation of lag 0 weighted by Gasteiger charge
Autocorrelation	GATS1Z	0.0169	Moreau–Broto autocorrelation of lag 1 weighted by atomic number
Autocorrelation	ATS5pe	0.0167	Moreau–Broto autocorrelation of lag 5 weighted by Pauling EN
TopoPSA	TopoPSA	0.0161	Topological polar surface area
Autocorrelation	ATS5se	0.0160	Moreau–Broto autocorrelation of lag 5 weighted by Sanderson EN
Autocorrelation	ATS4p	0.0150	Moreau–Broto autocorrelation of lag 4 weighted by polarizability
Autocorrelation	ATS4i	0.0144	Moreau–Broto autocorrelation of lag 4 weighted by ionization potential
Autocorrelation	GATS1are	0.0131	Geary coefficient of lag 1 weighted by Allred–Rocow EN
Autocorrelation	GATS1pe	0.0126	Geary coefficient of lag 1 weighted by Pauling EN
MoeType	VSA_EState3	0.0122	VSA EState Descriptor 3 (5.00 ≤ *x* < 5.41)
Autocorrelation	ATS5are	0.0122	Moreau–Broto autocorrelation of lag 5 weighted by Allred–Rocow EN
Autocorrelation	GATS1m	0.0122	Geary coefficient of lag 1 weighted by mass
Autocorrelation	ATSC1are	0.0115	Centered Moreau–Broto autocorrelation of lag 1 weighted by Allred–Rocow EN
Autocorrelation	ATSC1c	0.0112	Centered Moreau–Broto autocorrelation of lag 1 weighted by Gasteiger charge
InformationContent	BIC0	0.0111	0-ordered bonding information content
Autocorrelation	GATS2Z	0.0110	Geary coefficient of lag 2 weighted by atomic number
AtomCount	nAtom	0.0109	Molecular ID on O atoms

**Fig. 5:**
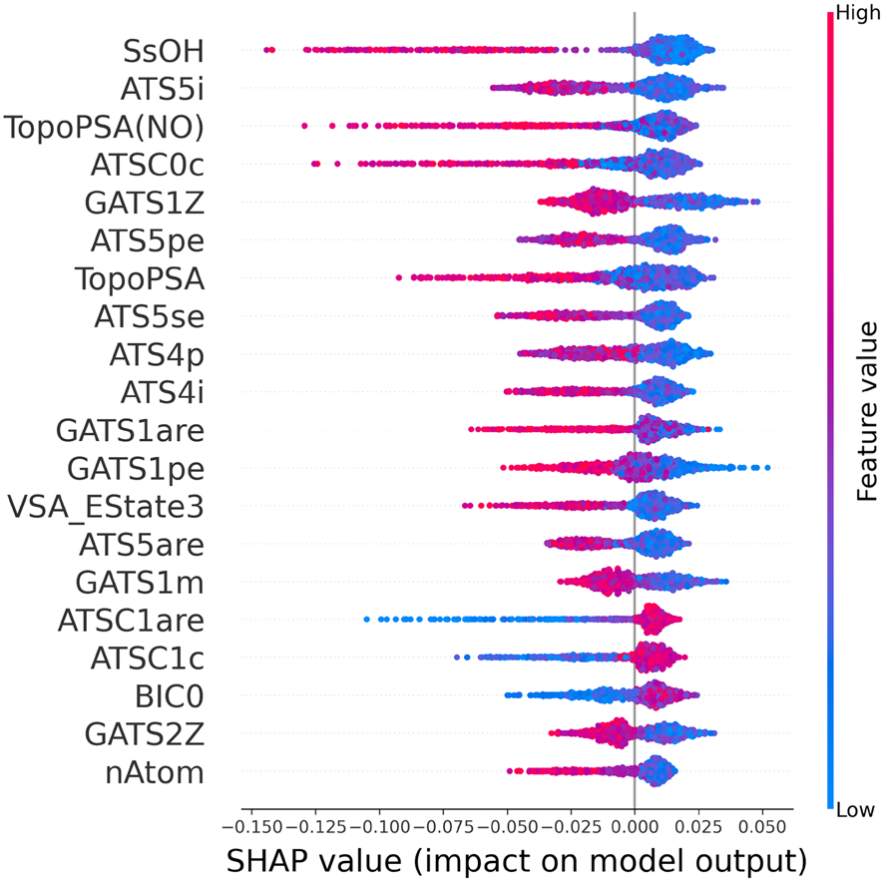
SHAP dependence plot of the top 20 features of the consensus model when the cutoff is 50%

The SHAP dependence plot is further used to understand how a single feature affects the output of the prediction models, where the color bar indicates the actual values of the features, and the SHAP values are plotted on the *x*-axis. The dependence plot of the consensus model when the cutoff is 50% was obtained by averaging the contributions of the individual models (Fig. 5, Additional file 1: Figs. S6–10). For each feature, a dot is created for each molecule, and positive SHAP values for a specific feature represent an increase in the predicted HOB value. For example, TopoPSA (NO) (topological polar surface area, using only nitrogen and oxygen) is an important feature that is overall negatively correlated with HOB (Fig. 5), i.e., a higher TopoPSA(NO) value will reduce the predicted HOB value. This is consistent with the finding that reducing the polar surface area increases the permeation rate of a molecule [32]. Similarly, the TopoPSA descriptor which calculates the entire polar surface area is also negatively correlated with the HOB value.

In addition, SsOH which is the total number of OH bonds also significantly affects the prediction of HOB. The blue dots are mainly concentrated in the area where the SHAP is greater than 0, therefore a small SsOH value will increase the HOB value. Decreasing the number of OH groups will increase the hydrophobicity and membrane absorption of a molecule, therefore leading to higher HOB. This is in line with the Lipinski's ‘Rule-of-Five’ [33]: if the number of hydrogen bond donors exceeds 5, the absorption or permeability may be poor [34].

It is believed that the charge state of molecules exerts a key influence on the perception of biomolecules (including membranes, enzymes and transporters) [35]. Several features that have great influence in the consensus model, such as RPCG in RF1, ATSC0c in RF2 and RNCG in RF4 are charge related descriptors.

Using the same method, we also analyzed the model obtained with the 20% cutoff, and obtained importance matrix plots (Additional file 1: Figs. S11–S15, S21) and the dependence plots (Additional file 1: Figs. S16–S20, S22) of the individual and the consensus models. The important features from the models trained with the two cutoffs are overall consistent, e.g., *TopoPSA (NO)*, *SsOH* and *Autocorrelation* also have a significant impact on the F = 20% consensus model as that on the F = 50% model.

## Discussion and conclusions

On the basis of a comprehensive data sets collected in this study, accurate RF models for prediction of HOB were developed. Moreover, we also analyzed the importance of the features, and found that the number of OH bonds and polar surface area of a molecule are negatively correlated with the HOB value, which is consistent with molecular characteristics that affect the oral drug bioavailability. The model is available as a web server at www.icdrug.com/ICDrug/ADMET, which provides researchers with an accurate tool to quickly and automatically predict the HOB of new molecules without any machine learning or statistical modeling knowledge.

## Supplementary Information

**Additional file 1: Table S1.** Cut-off values used in different studies and the number of positive and negative samples in each study. **Table S2.** List of descriptors calculated using Mordred. **Table S3.** The performance of the consensus model on the training set and each fold in the fivefold cross validation when the cutoff is 50%. **Table S4.** The performance of the consensus model on the training set and each fold in fivefold cross validation when the cutoff is 20%. **Figure S1.** The importance matrix plot for the RF model 1 when the cutoff is 50%. **Figure S2.** The importance matrix plot for the RF model 2 when the cutoff is 50%. **Figure S3.** The importance matrix plot for the RF model 3 when the cutoff is 50%. **Figure S4.** The importance matrix plot for the RF model 4 when the cutoff is 50%. **Figure S5.** The importance matrix plot for the RF model 5 when the cutoff is 50%. **Figure S6.** SHAP dependence plot of the top 20 features of the RF model 1 when the cutoff is 50%. **Figure S7.** SHAP dependence plot of the top 20 features of the RF model 2 when the cutoff is 50%. **Figure S8.** SHAP dependence plot of the top 20 features of the RF model 3 when the cutoff is 50%. **Figure S9.** SHAP dependence plot of the top 20 features of the RF model 4 when the cutoff is 50%. **Figure S10.** SHAP dependence plot of the top 20 features of the RF model 5 when the cutoff is 50%. **Figure S11.** The importance matrix plot for the RF model 1 when the cutoff is 20%. **Figure S12.** The importance matrix plot for the RF model 2 when the cutoff is 20%. **Figure S13.** The importance matrix plot for the RF model 3 when the cutoff is 20%. **Figure S14.** The importance matrix plot for the RF model 4 when the cutoff is 20%. **Figure S15.** The importance matrix plot for the RF model 5 when the cutoff is 20%. **Figure S16.** SHAP dependence plot of the top 20 features of the RF model 1 when the cutoff is 20%. **Figure S17.** SHAP dependence plot of the top 20 features of the RF model 2 when the cutoff is 20%. **Figure S18.** SHAP dependence plot of the top 20 features of the RF model 3 when the cutoff is 20%. **Figure S19.** SHAP dependence plot of the top 20 features of the RF model 4 when the cutoff is 20%. **Figure S20.** SHAP dependence plot of the top 20 features of the RF model 5 when the cutoff is 20%. **Figure S21.** (A) Importance matrix plot of the consensus model when the cutoff is 20%. (B) A statistical graph of the number of occurrences of the top 20 features that affect all models when the cutoff is 20%. **Figure S22.** SHAP dependence plot of the top 20 features of the consensus model when the cutoff is 20%.

## Data Availability

The training and test data sets and the trained models are available at https://github.com/whymin/HOB and the web server at www.icdrug.com/ICDrug/ADMET. Other data are available from the corresponding author on reasonable request.

## Abbreviations

HOB: Human oral bioavailability
ADMET: Absorption, distribution, metabolism, excretion, and toxicity
QSPR: Quantitative structure property relationships
SHAP: The SHapley Additive exPlanation
SE: Sensitivity
SP: Specificity
ACC: Accuracy of prediction
MCC: Matthew’s correlation coefficient
ROC: The receiver operating characteristic
AUC: The receiver operating characteristic curve and the area under the curve
RF: Random forest
CART: Classification and regression trees
MLP: Multilayer perceptron
NB: Naive Bayes
GBT: Gradient boosted trees
SVM: Support vector machines
PCA: Principal component analysis
