# Supplementary material for: HobPre: accurate prediction of human oral bioavailability for small molecules
Source: J Cheminform. 2022 Jan 6;14:1. doi: 10.1186/s13321-021-00580-6 (PMC8740492; doi:10.1186/s13321-021-00580-6)
Supplement: Supplementary file 1 — Additional file 1: Table S1. Cut-off values used in different studies and the number of positive and negative samples in each study. Table S2. List of descriptors calculated using Mordred. Table S3. The performance of the consensus model on the training set and each fold in the fivefold cross validation when the cutoff is 50%. Table S4. The performance of the consensus model on the training set and each fold in fivefold cross validation when the cutoff is 20%. Figure S1. The importance matrix plot for the RF model 1 when the cutoff is 50%. Figure S2. The importance matrix plot for the RF model 2 when the cutoff is 50%. Figure S3. The importance matrix plot for the RF model 3 when the cutoff is 50%. Figure S4. The importance matrix plot for the RF model 4 when the cutoff is 50%. Figure S5. The importance matrix plot for the RF model 5 when the cutoff is 50%. Figure S6. SHAP dependence plot of the top 20 features of the RF model 1 when the cutoff is 50%. Figure S7. SHAP dependence plot of the top 20 features of the RF model 2 when the cutoff is 50%. Figure S8. SHAP dependence plot of the top 20 features of the RF model 3 when the cutoff is 50%. Figure S9. SHAP dependence plot of the top 20 features of the RF model 4 when the cutoff is 50%. Figure S10. SHAP dependence plot of the top 20 features of the RF model 5 when the cutoff is 50%. Figure S11. The importance matrix plot for the RF model 1 when the cutoff is 20%. Figure S12. The importance matrix plot for the RF model 2 when the cutoff is 20%. Figure S13. The importance matrix plot for the RF model 3 when the cutoff is 20%. Figure S14. The importance matrix plot for the RF model 4 when the cutoff is 20%. Figure S15. The importance matrix plot for the RF model 5 when the cutoff is 20%. Figure S16. SHAP dependence plot of the top 20 features of the RF model 1 when the cutoff is 20%. Figure S17. SHAP dependence plot of the top 20 features of the RF model 2 when the cutoff is 20%. Figure S18. SHAP dependence plot of th [file 13321_2021_580_MOESM1_ESM.docx]

**ADDITIONAL INFORMATION**

**HobPre: accurate prediction of human oral bioavailability for small molecules**

Min Wei^1^, Xudong Zhang^1^, Xiaolin Pan^1^, Bo Wang^1^, Changge Ji^1,2*^, Yifei Qi^4*^, and John Z.H. Zhang^1,2,3*^

*^1^Shanghai Engineering Research Center of Molecular Therapeutics & New Drug Development, Shanghai Key Laboratory of Green Chemistry & Chemical Process, School of Chemistry and Molecular Engineering, East China Normal University, Shanghai 200062, China*

*^2^NYU-ECNU Center for Computational Chemistry at NYU Shanghai, Shanghai 200062, China
^3^Department of Chemistry, New York University, NY, NY 10003, USA*

*^4^Department of Medicinal Chemistry, School of Pharmacy, Fudan University, Shanghai 201203, China*

**Table S1**. Cut-off values used in different studies and the number of positive and negative samples in each study.

| Cut-off value | Year | Model | Positive | Negative |
| --- | --- | --- | --- | --- |
| F=20% | 2008 | Ma et al.^14^ | 690 | 76 |
|  | 2018 | ADMETlab^15^ | 759 | 254 |
| F=30% | 2018 | ADMETlab^15^ | 672 | 341 |
| **F=50%** | 2020 | Falcón-Cano et al.^7^ | 621 | 573 |
|  | 2019 | admetSAR^8^ | 509 | 486 |
|  | 2014 | Kim et al.^9^ | 540 | 455 |
| F=80% | 2012 | Ahmed et al.^5^ | 293 | 676 |

***Table S2****. List of descriptors calculated using Mordred.*

| Descriptor category | Number of descriptors (all) | Number of descriptors  (reduced) |
| --- | --- | --- |
| ABCIndex | 2 | 2 |
| AcidBase | 2 | 2 |
| AdjacencyMatrix | 13 | 12 |
| Aromatic | 2 | 2 |
| AtomCount | 16 | 17 |
| Autocorrelation | 606 | 465 |
| BCUT^a^ | 24 | 24 |
| BalabanJ^a^ | 1 | 1 |
| BaryszMatrix^a^ | 104 | 104 |
| BertzCT | 1 | 1 |
| BondCount | 9 | 9 |
| CarbonTypes | 11 | 11 |
| Chi | 56 | 48 |
| Constitutional | 16 | 16 |
| DetourMatrix | 14 | 0 |
| DistanceMatrix | 13 | 12 |
| Estate | 316 | 82 |
| EccentricConnectivityIndex | 1 | 1 |
| ExtendedTopochemicalAtom | 45 | 45 |
| FragmentComplexity | 1 | 1 |
| Framework | 1 | 1 |
| HydrogenBond^a^ | 2 | 2 |
| InformationContent | 42 | 42 |
| KappaShapeIndex | 3 | 3 |
| Lipinski | 2 | 2 |
| LogS | 1 | 1 |
| McGowanVolume | 1 | 1 |
| MoeType^a^ | 53 | 51 |
| MolecularDistanceEdge | 19 | 0 |
| MolecularId | 12 | 12 |
| PathCount | 21 | 21 |
| Polarizability | 2 | 2 |
| RingCount | 138 | 87 |
| RotatableBond^a^ | 2 | 2 |
| SLogP^a^ | 2 | 2 |
| TopoPSA^a^ | 2 | 2 |
| TopologicalCharge | 21 | 21 |
| TopologicalIndex | 4 | 4 |
| VdwVolumeABC | 1 | 0 |
| VertexAdjacencyInformation | 1 | 1 |
| WalkCount | 21 | 21 |
| Weight | 2 | 2 |
| WienerIndex | 2 | 2 |
| ZagrebIndex | 4 | 4 |
| CPSA | 43 | 2 |
| Total | 1614 | 1143 |

**Table S3.** The performance of the consensus model on the training set and each fold in the 5-fold cross validation when the cutoff is 50%.

| Data | SE | SP | ACC | AUC | MCC | F1-score |
| --- | --- | --- | --- | --- | --- | --- |
| Training set | 0.907 | 0.957 | 0.934 | 0.985 | 0.868 | 0.940 |
| Fold 1 | 0.876 | 0.968 | 0.926 | 0.986 | 0.853 | 0.935 |
| Fold 2 | 0.881 | 0.975 | 0.931 | 0.978 | 0.864 | 0.937 |
| Fold 3 | 0.910 | 0.958 | 0.935 | 0.988 | 0.871 | 0.938 |
| Fold 4 | 0.927 | 0.951 | 0.939 | 0.988 | 0.878 | 0.943 |
| Fold 5 | 0.951 | 0.930 | 0.939 | 0.987 | 0.878 | 0.945 |

**Table S4**. The performance of the consensus model on the training set and each fold in 5-fold cross validation when the cutoff is 20%.

| Data | SE | SP | ACC | AUC | MCC | F1-score |
| --- | --- | --- | --- | --- | --- | --- |
| Training set | 0.972 | 0.981 | 0.979 | 0.997 | 0.944 | 0.986 |
| Fold 1 | 0.945 | 0.983 | 0.974 | 0.995 | 0.928 | 0.983 |
| Fold 2 | 0.980 | 0.989 | 0.987 | 0.999 | 0.963 | 0.992 |
| Fold 3 | 0.951 | 0.976 | 0.969 | 0.995 | 0.922 | 0.979 |
| Fold 4 | 1 | 0.982 | 0.983 | 0.995 | 0.958 | 0.988 |
| Fold 5 | 0.981 | 0.981 | 0.982 | 0.999 | 0.952 | 0.988 |


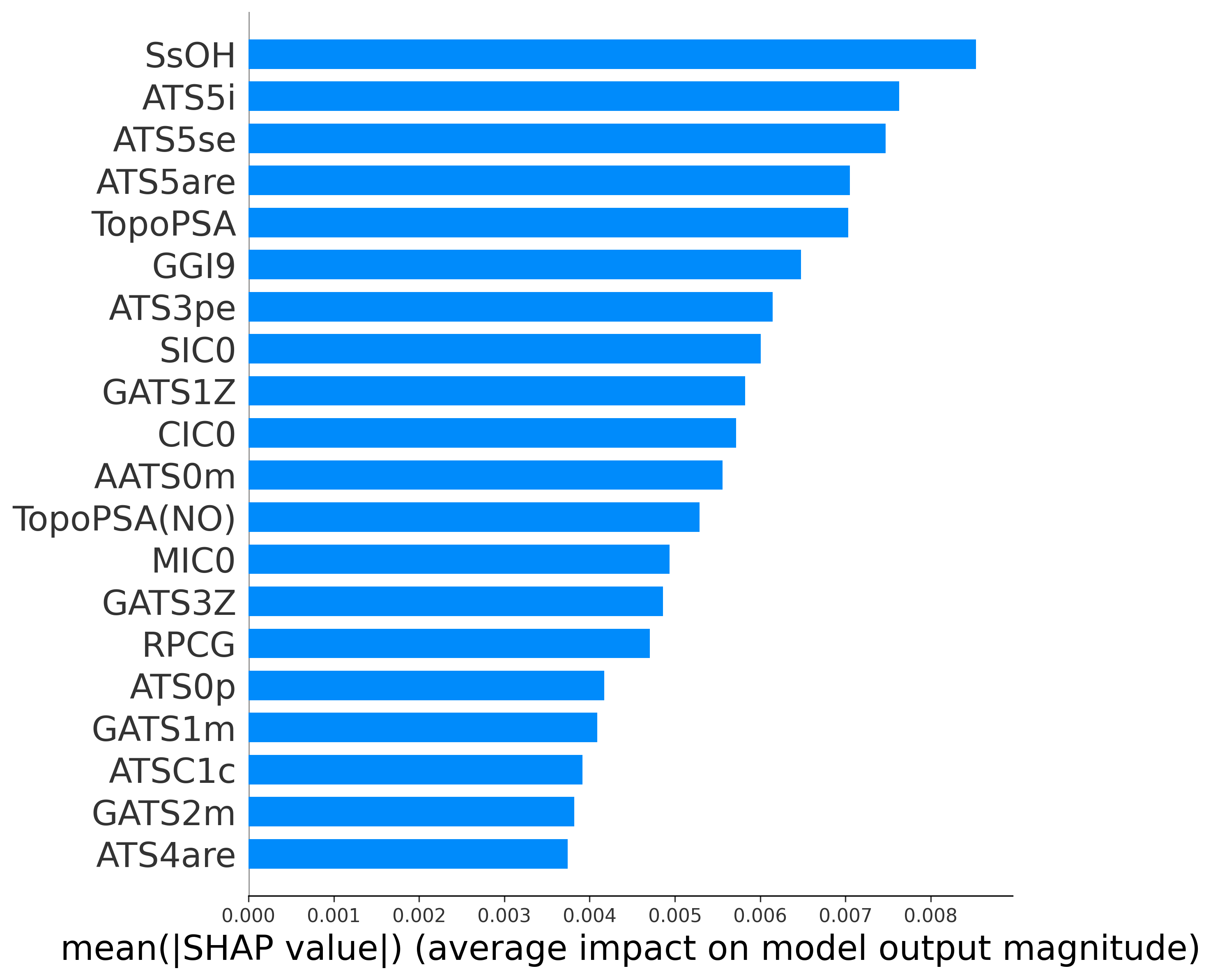


**Figure S1**. The importance matrix plot for the RF model 1 when the cutoff is 50%.


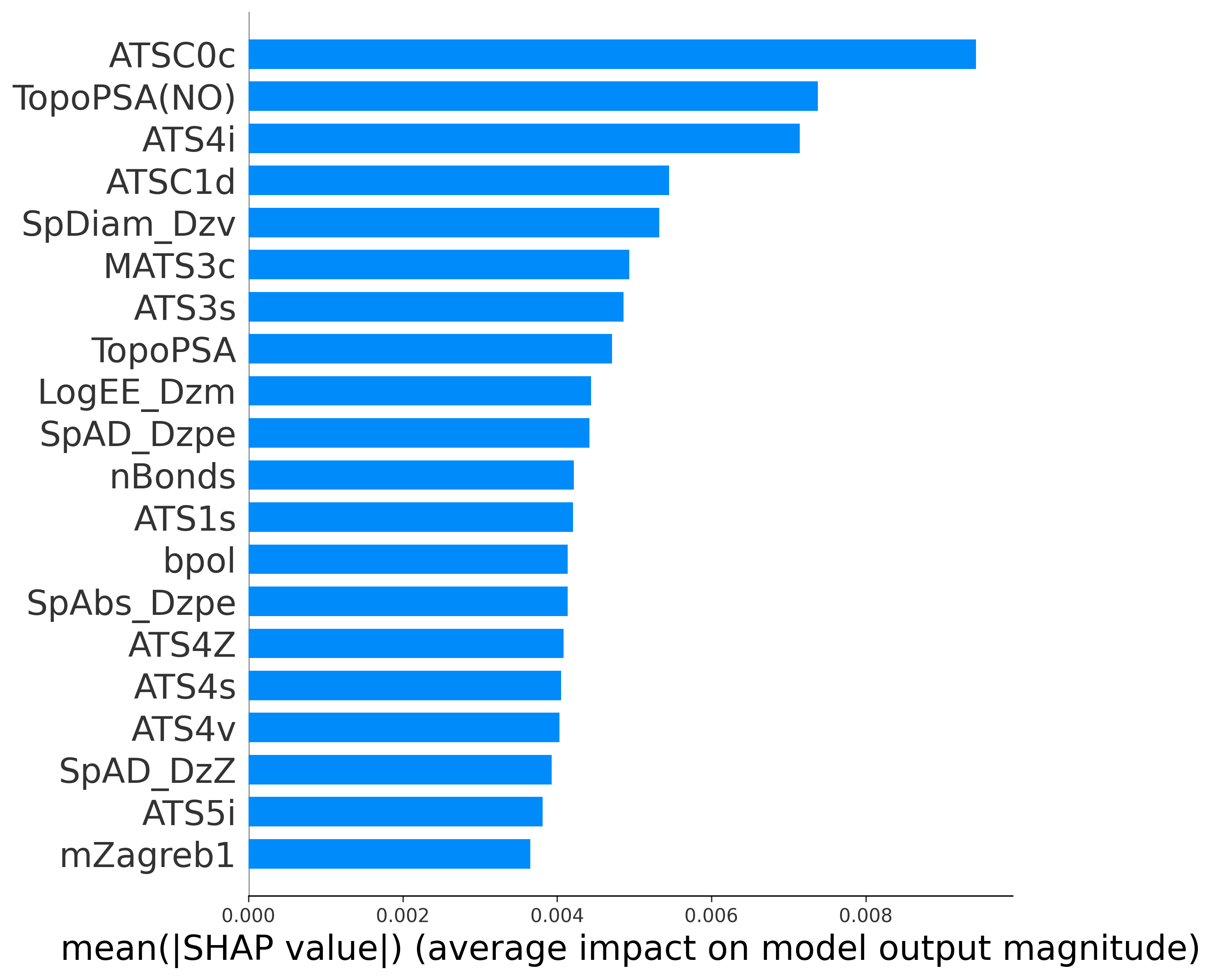


**Figure S2**. The importance matrix plot for the RF model 2 when the cutoff is 50%.


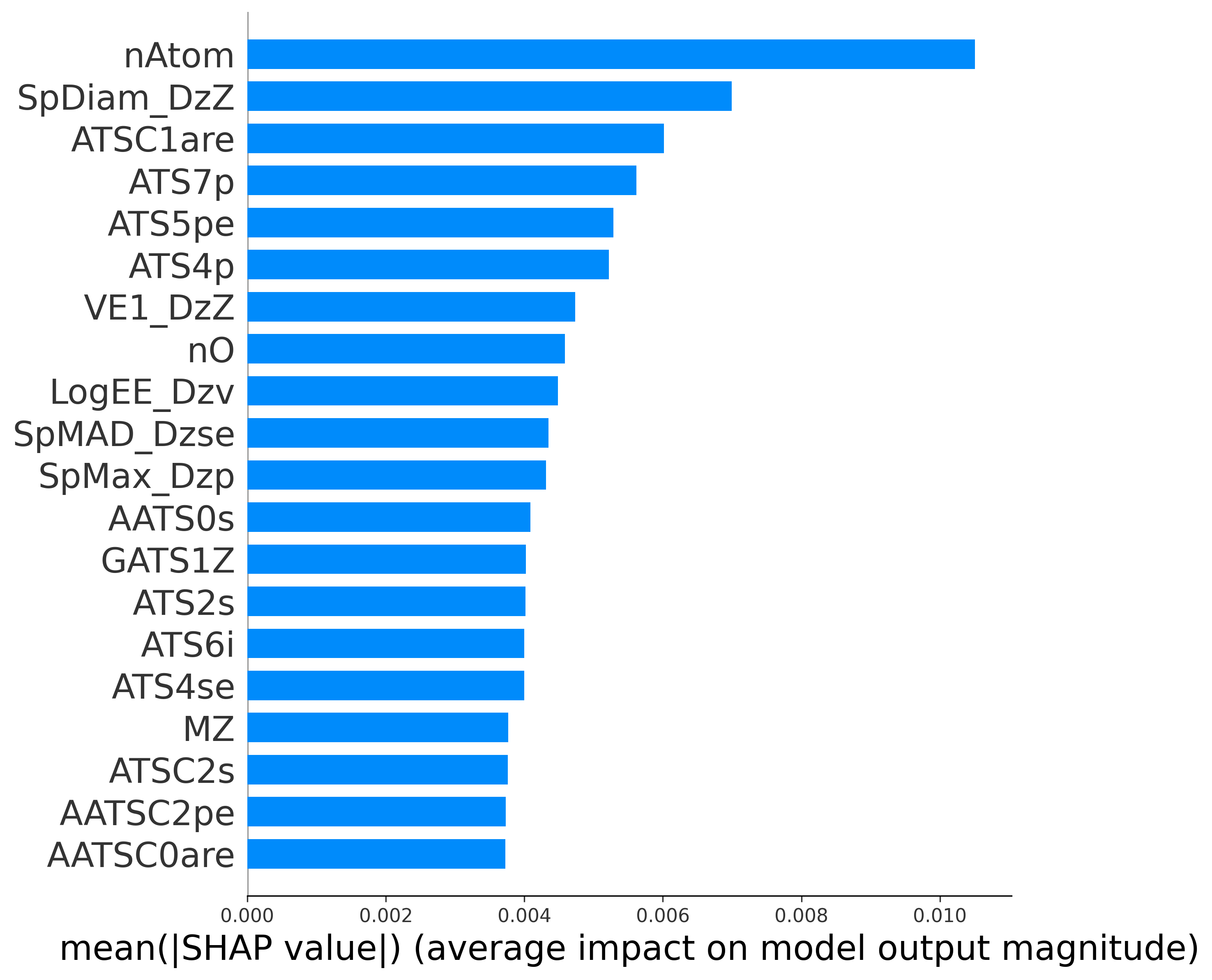


**Figure S3**. The importance matrix plot for the RF model 3 when the cutoff is 50%.


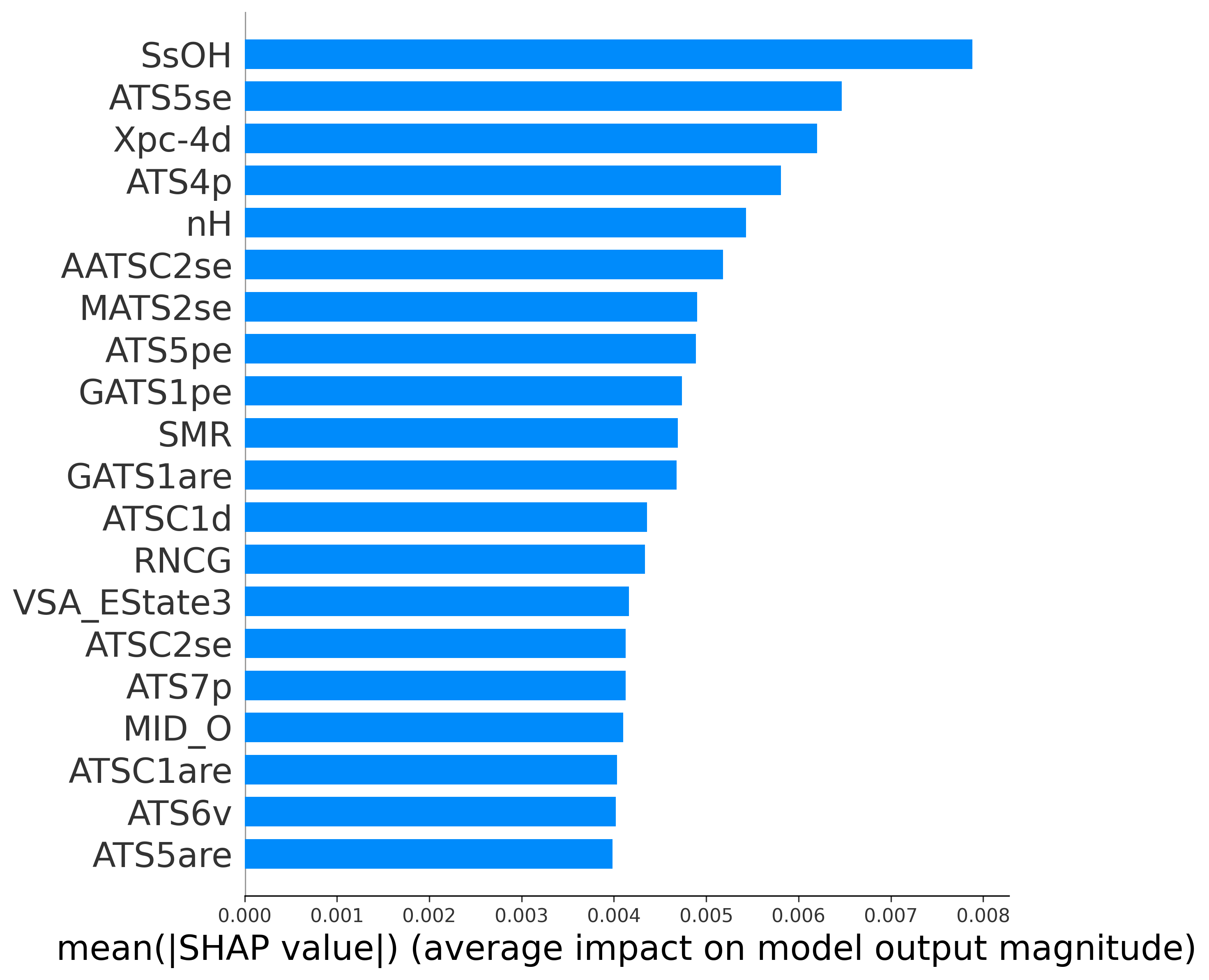


**Figure S4**. The importance matrix plot for the RF model 4 when the cutoff is 50%.


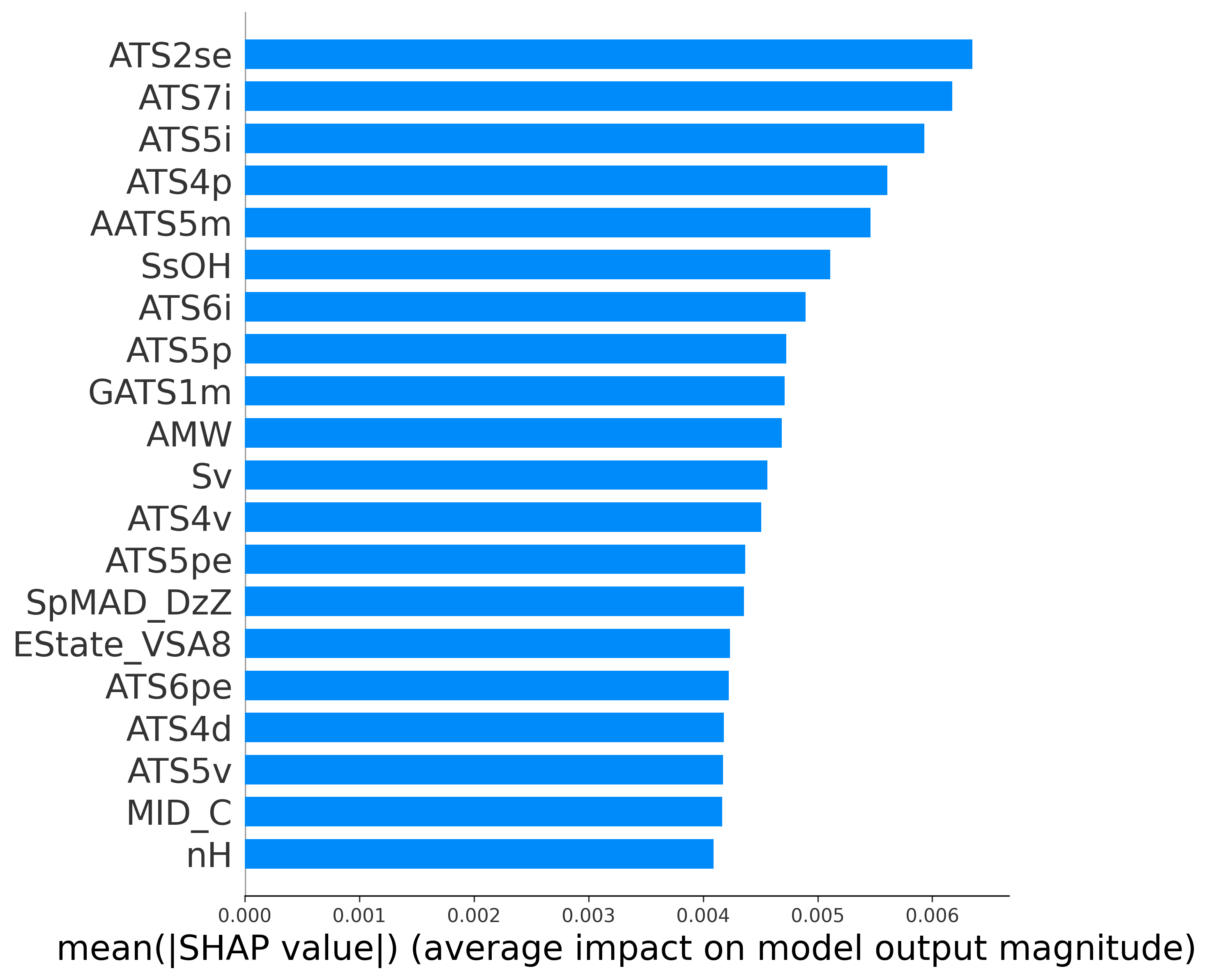


**Figure S5**. The importance matrix plot for the RF model 5 when the cutoff is 50%.


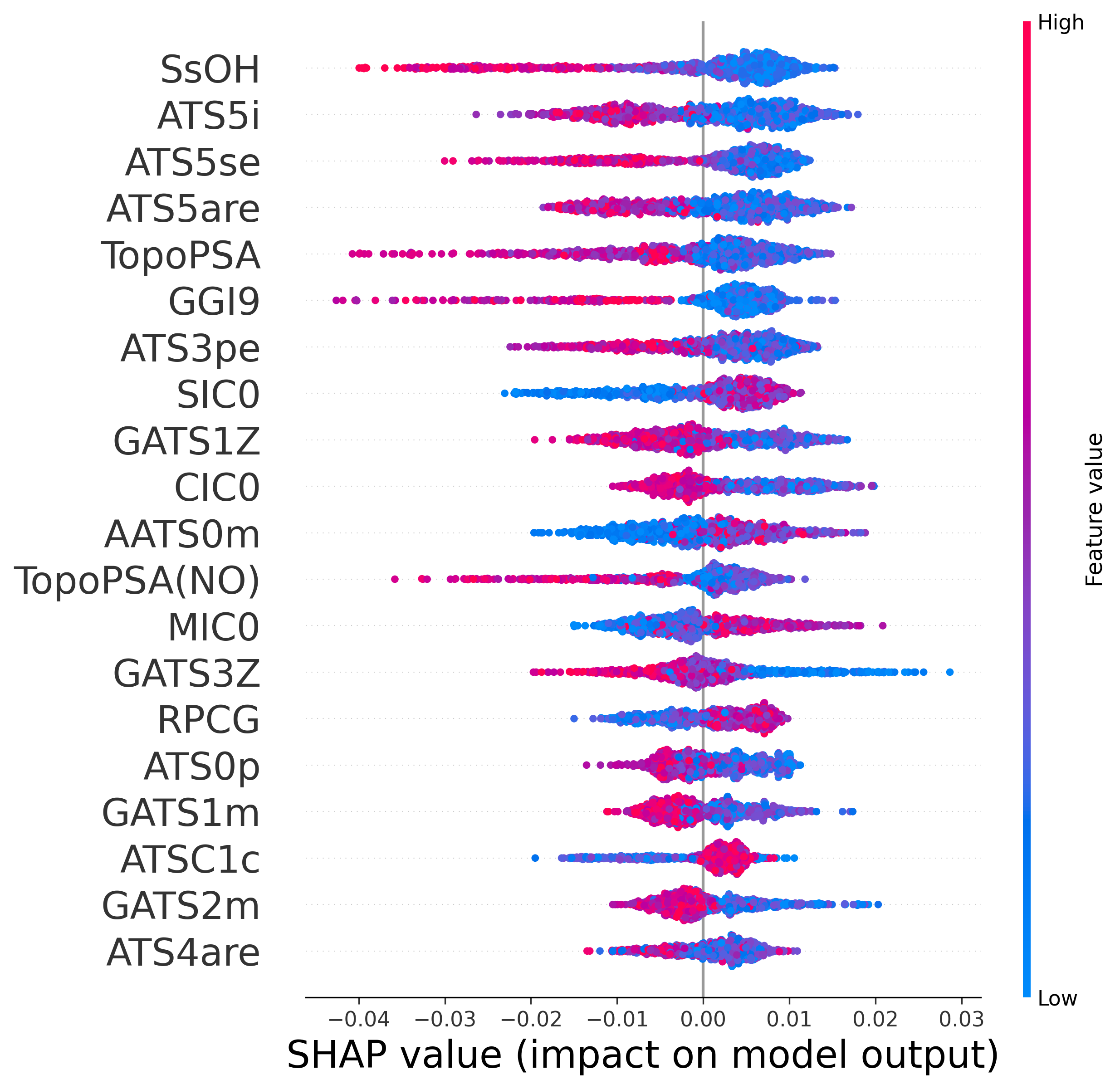


**Figure S6**. SHAP dependence plot of the top 20 features of the RF model 1 when the cutoff is 50%.


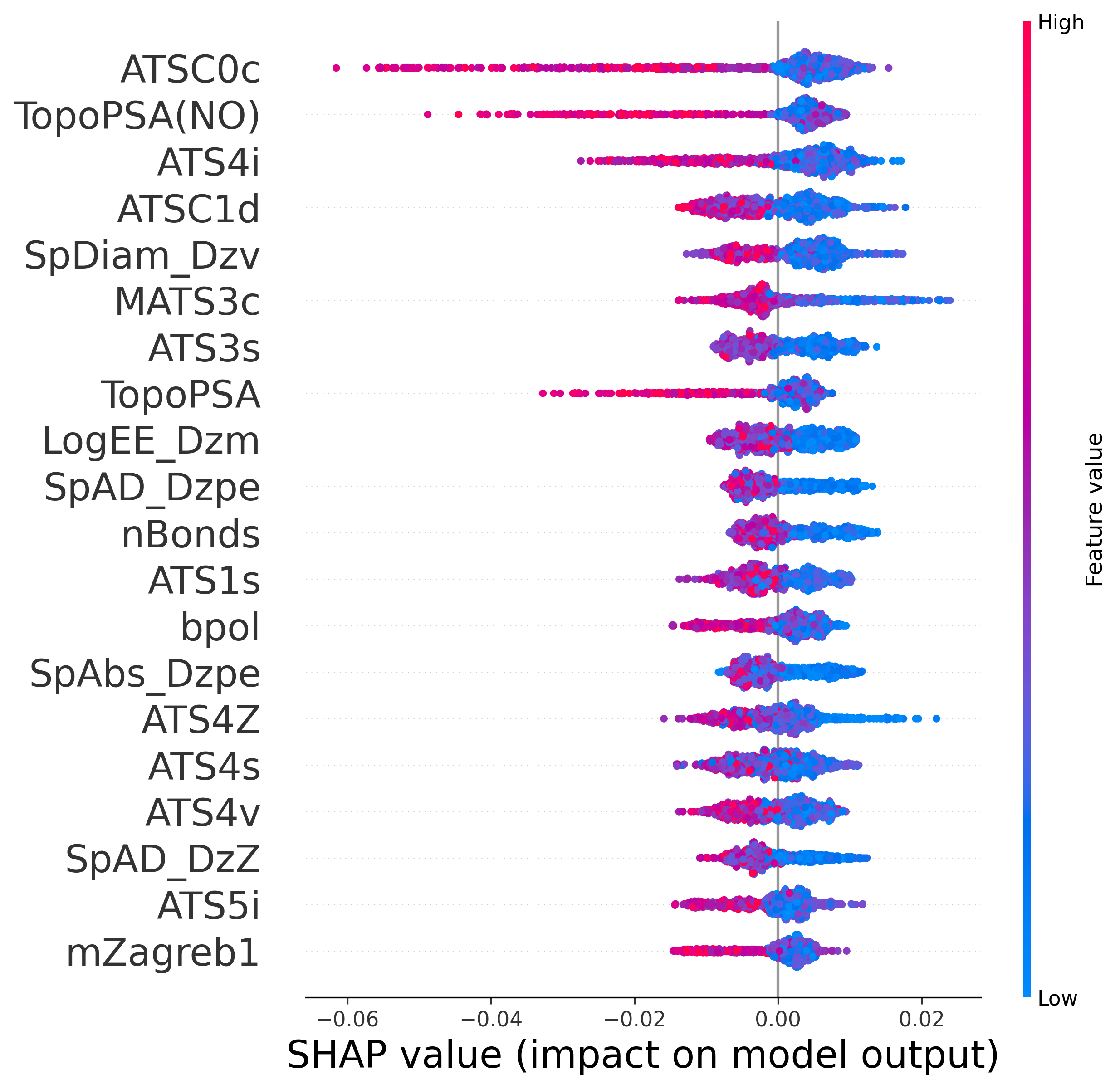


**Figure S7**. SHAP dependence plot of the top 20 features of the RF model 2 when the cutoff is 50%.


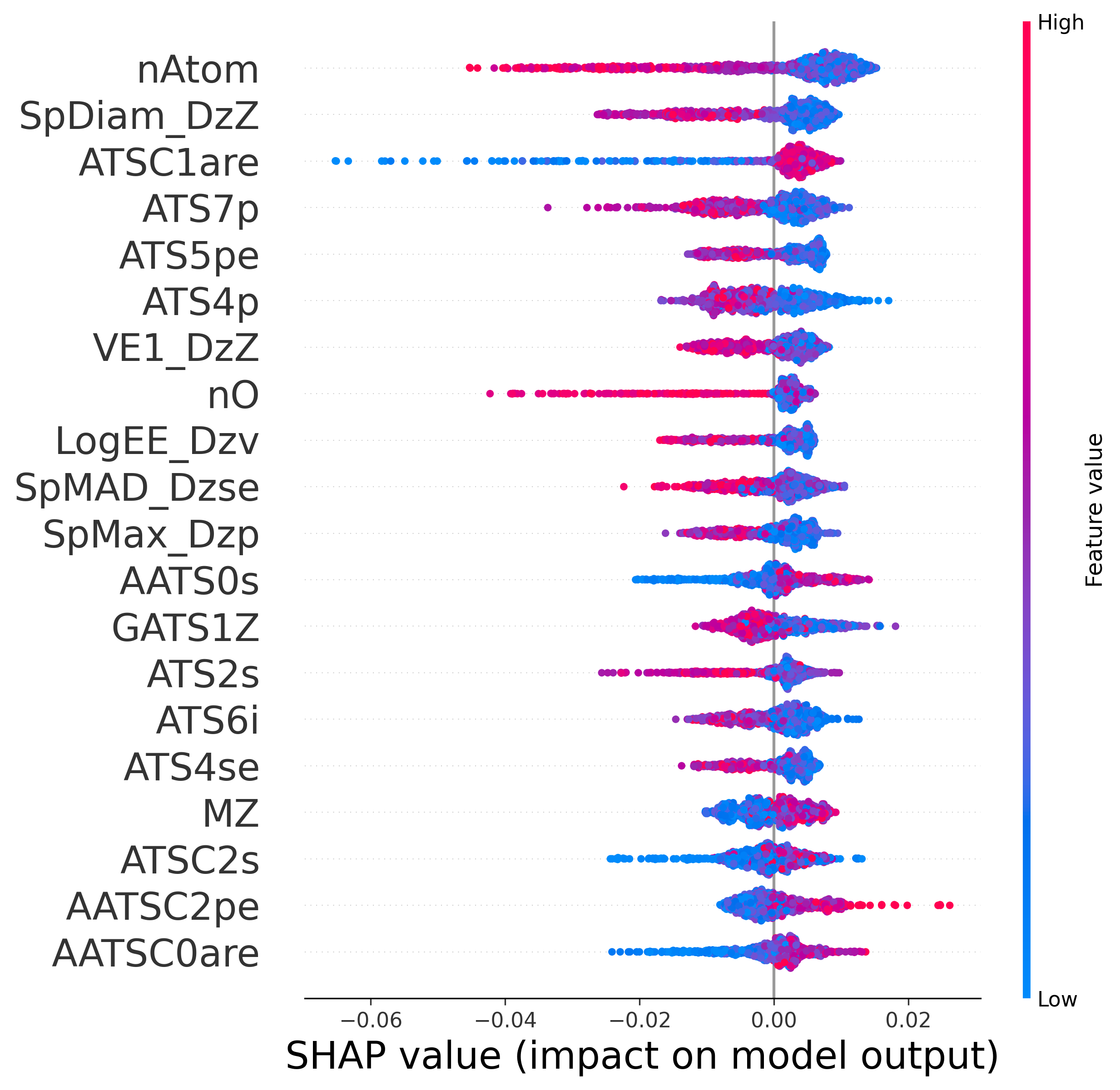


**Figure S8**. SHAP dependence plot of the top 20 features of the RF model 3 when the cutoff is 50%.


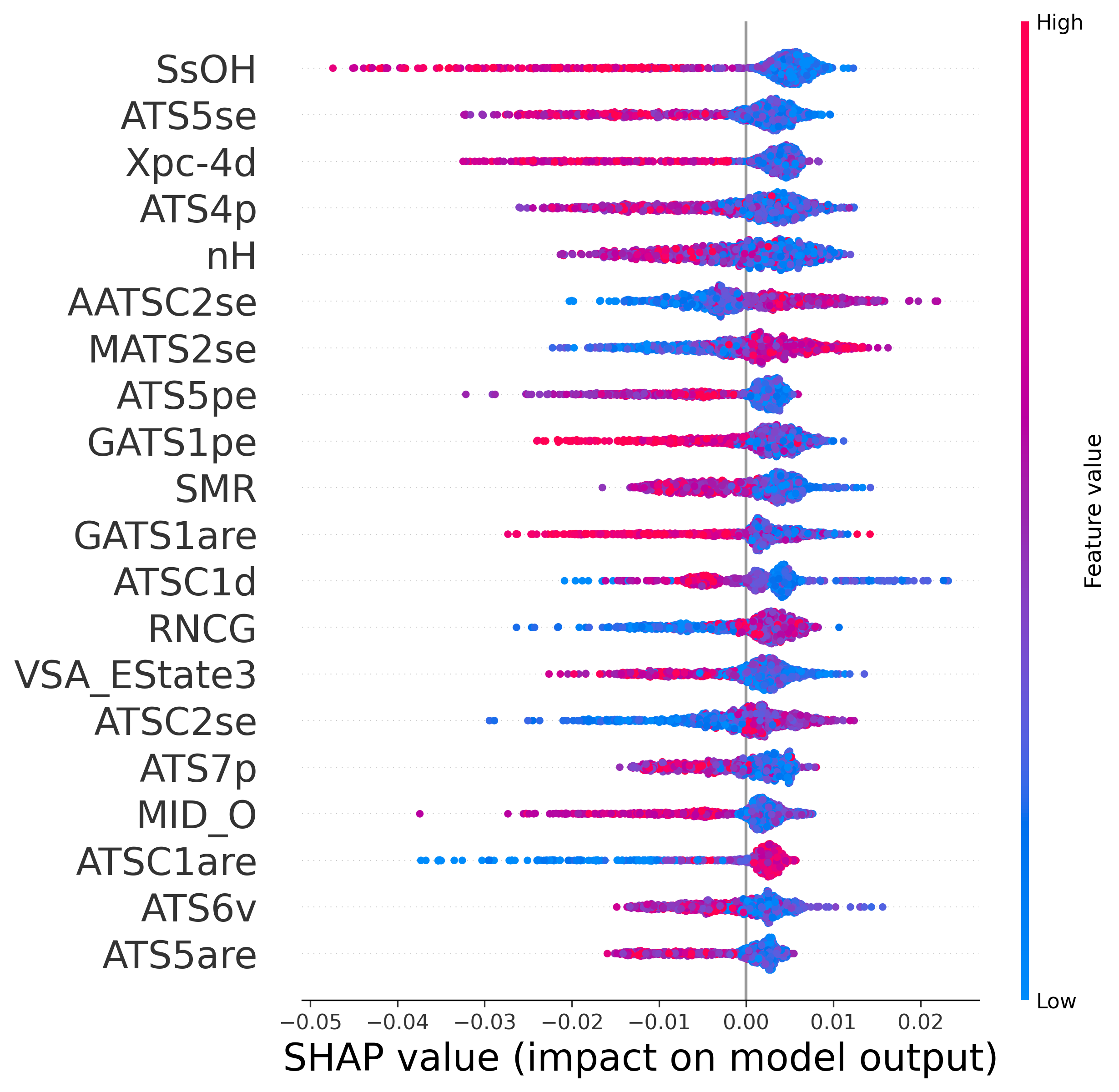


**Figure S9**. SHAP dependence plot of the top 20 features of the RF model 4 when the cutoff is 50%.


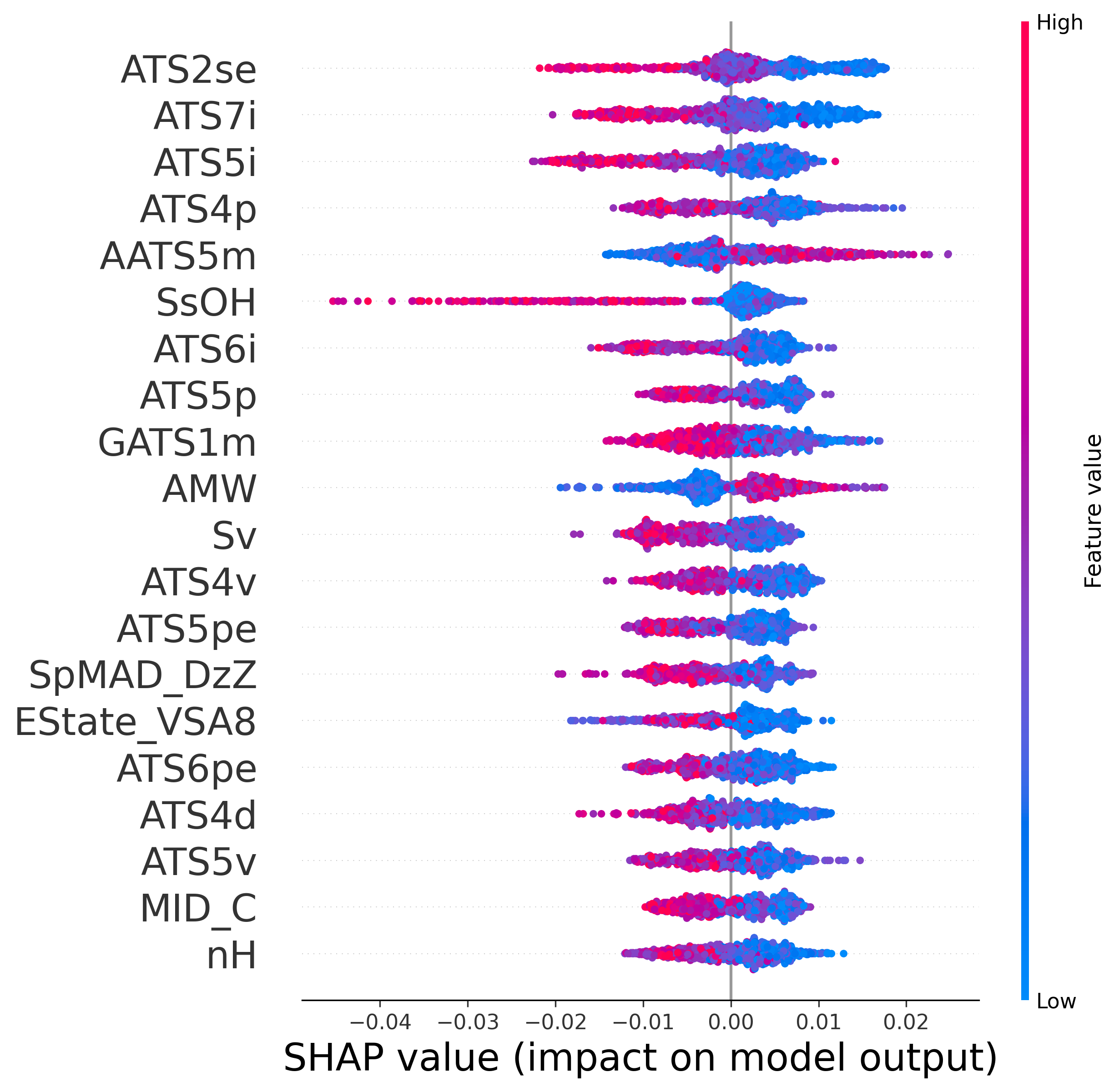


**Figure S10**. SHAP dependence plot of the top 20 features of the RF model 5 when the cutoff is 50%.


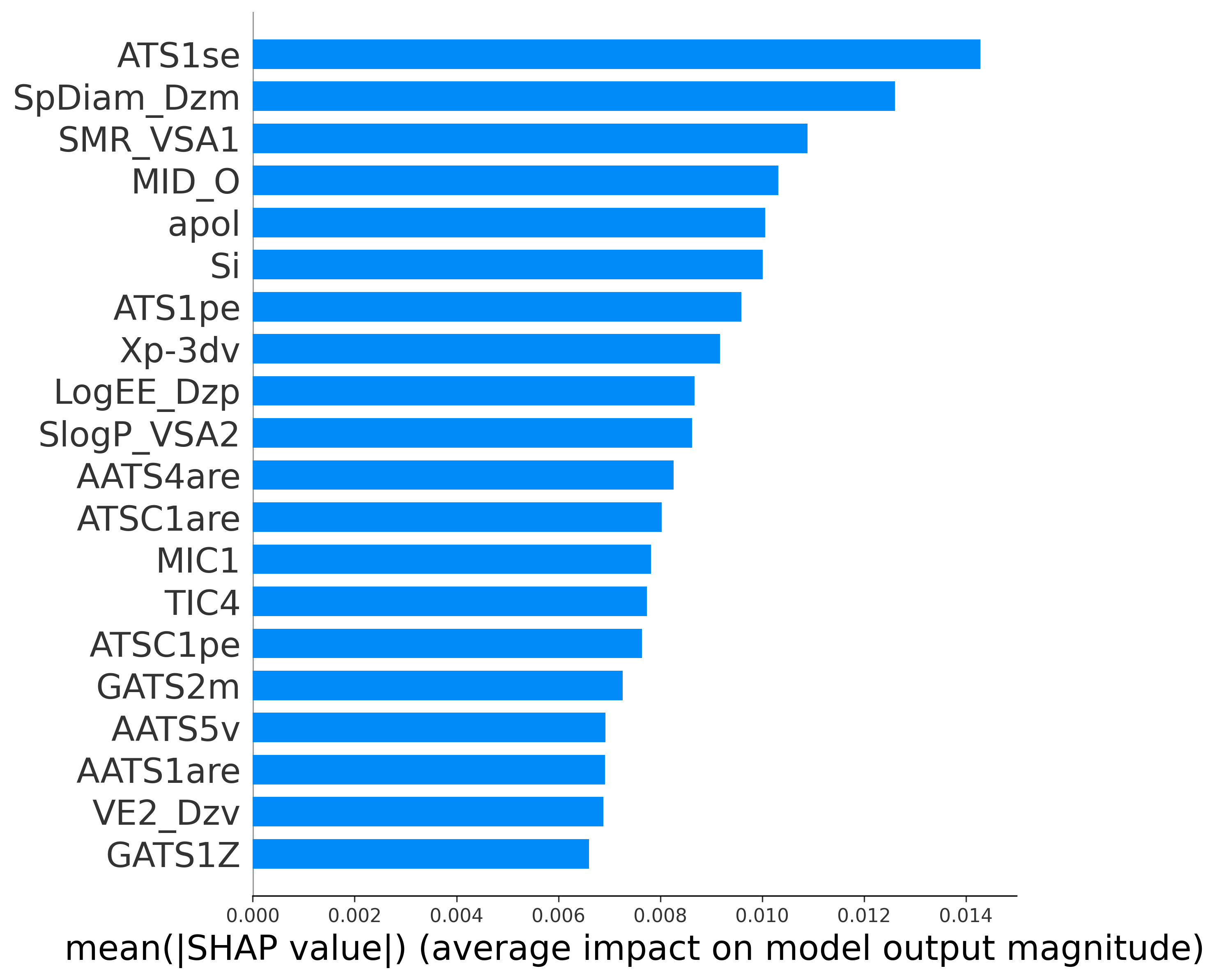


**Figure S11**. The importance matrix plot for the RF model 1 when the cutoff is 20%.


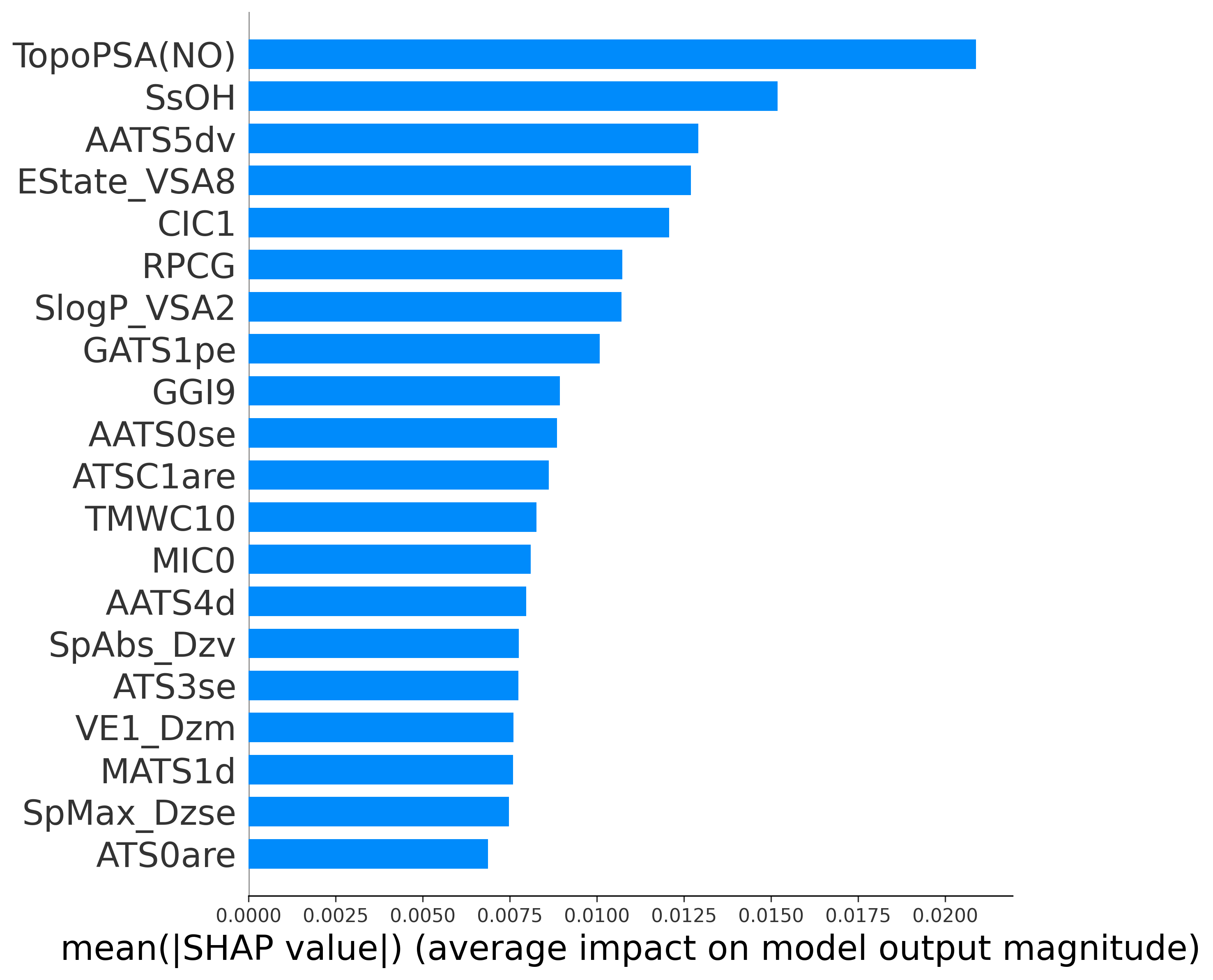


**Figure S12**. The importance matrix plot for the RF model 2 when the cutoff is 20%.


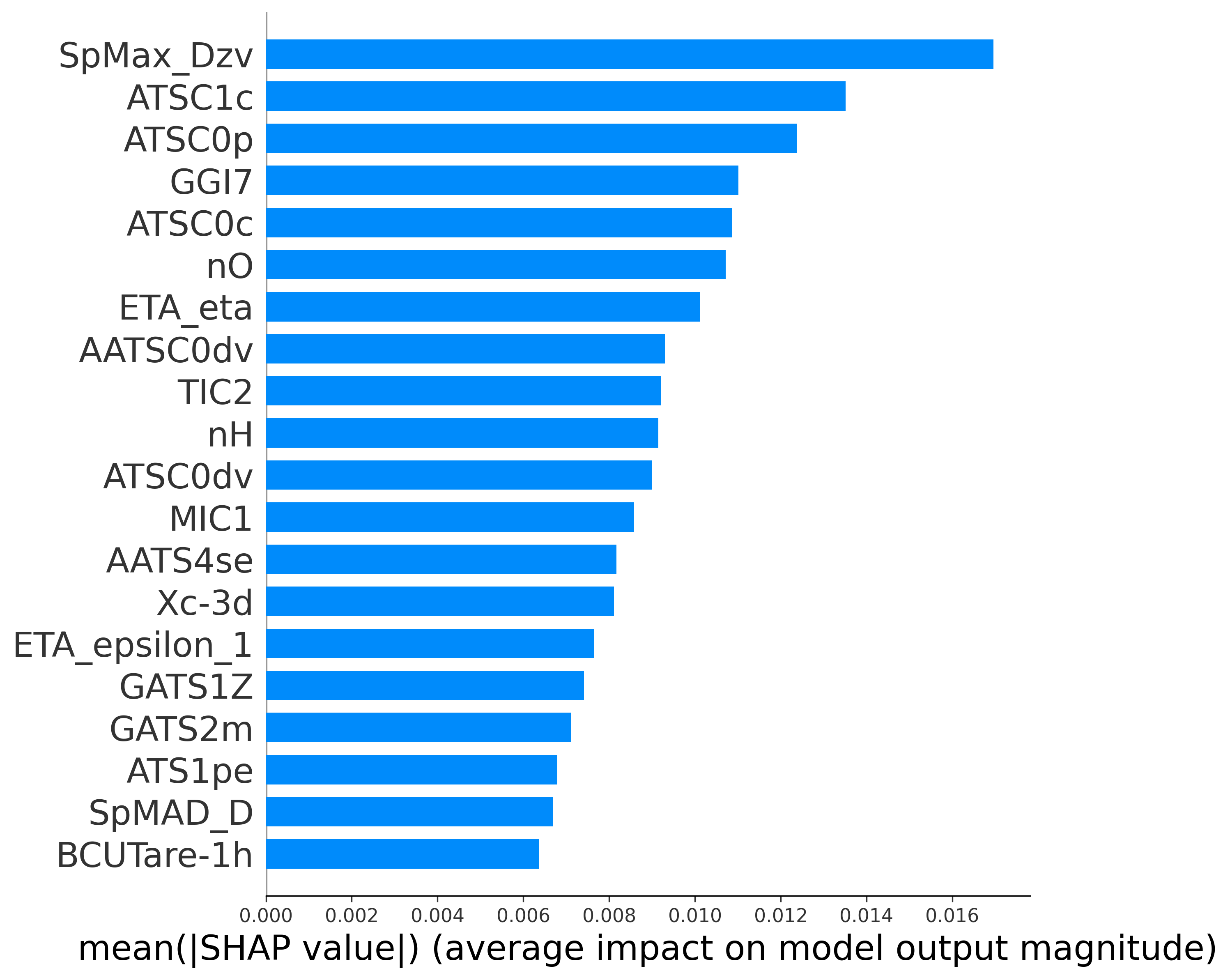


**Figure S13**. The importance matrix plot for the RF model 3 when the cutoff is 20%.


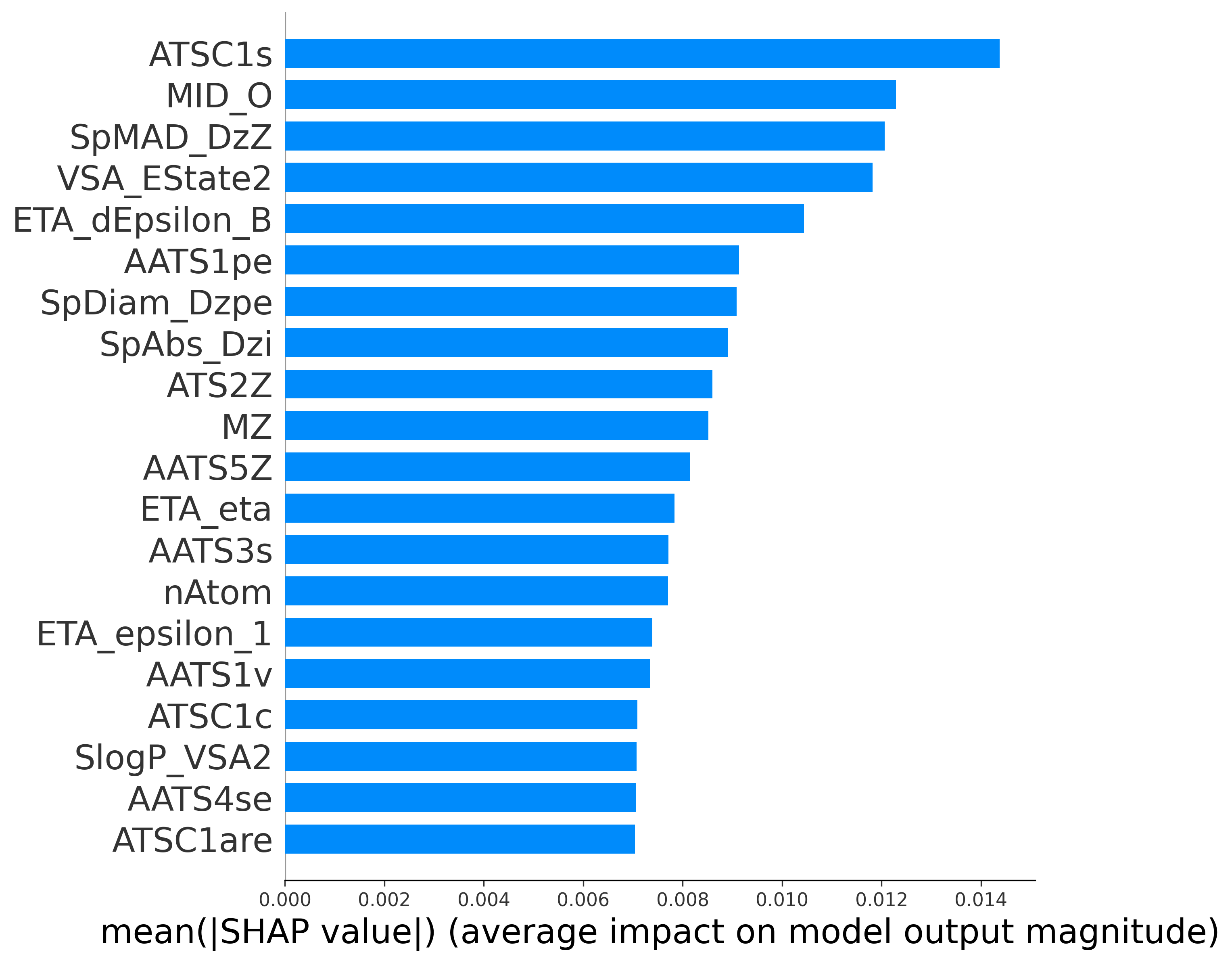


**Figure S14**. The importance matrix plot for the RF model 4 when the cutoff is 20%.


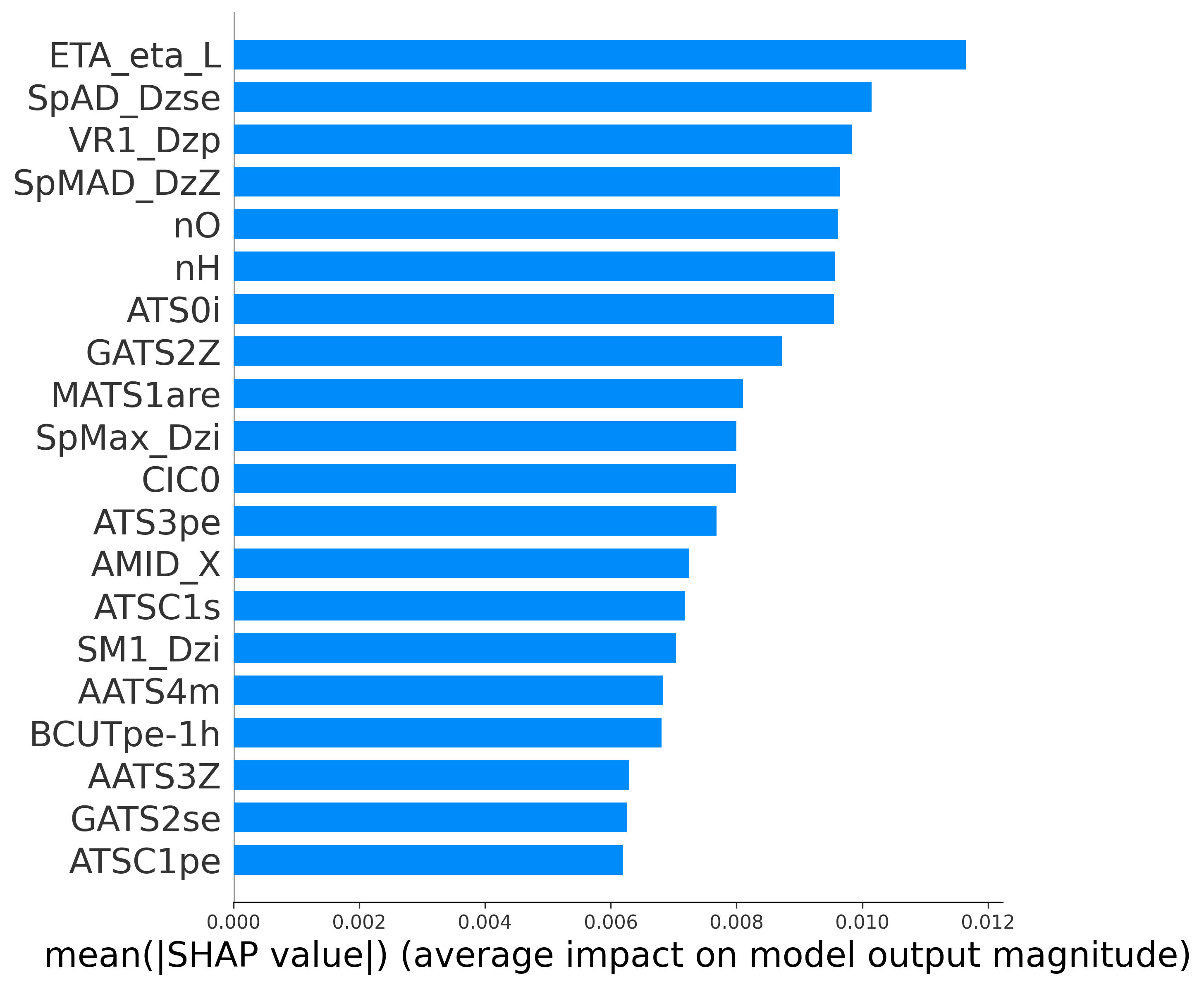
 **Figure S15**. The importance matrix plot for the RF model 5 when the cutoff is 20%.


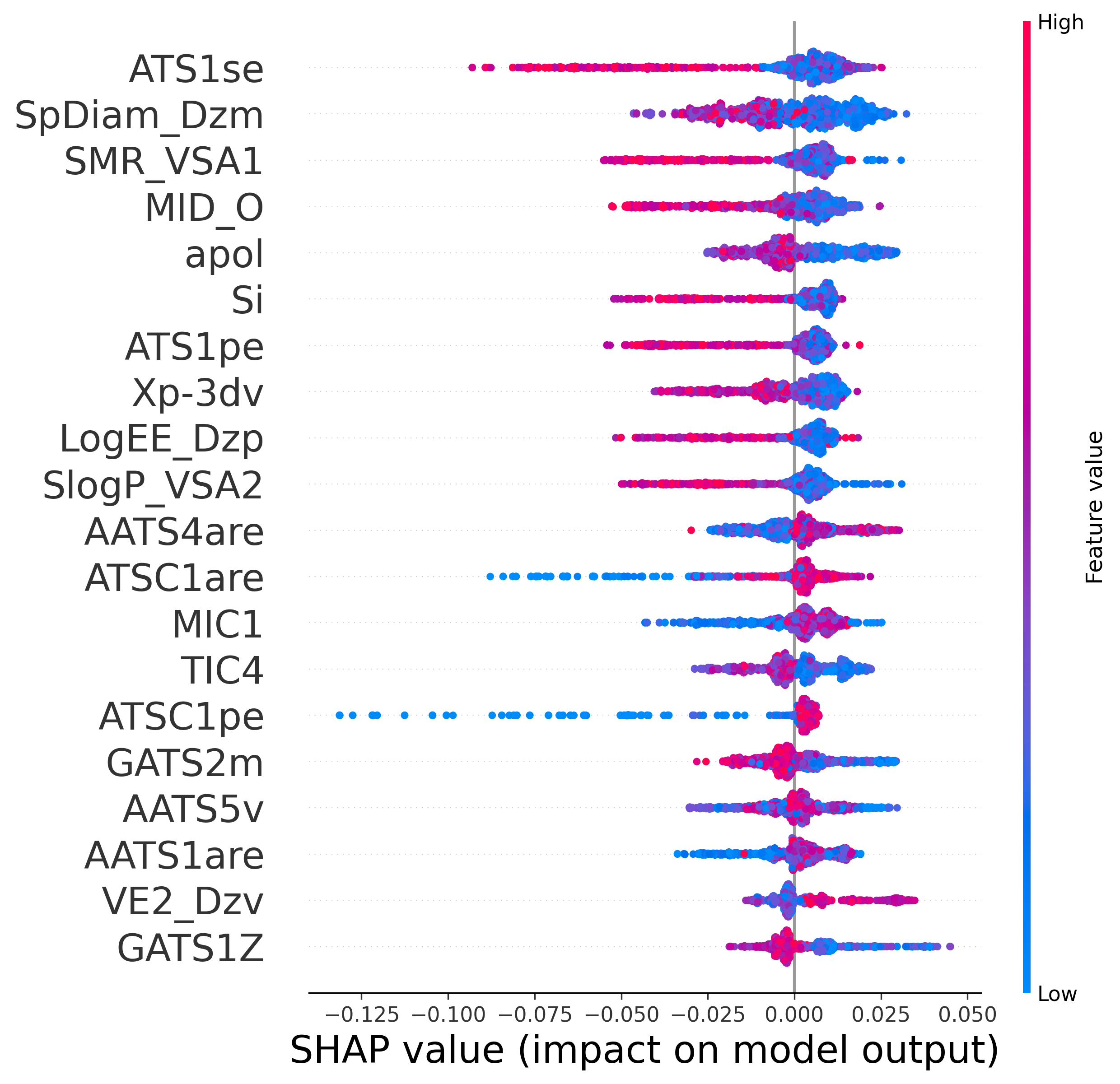
 **Figure S16**. SHAP dependence plot of the top 20 features of the RF model 1 when the cutoff is 20%.


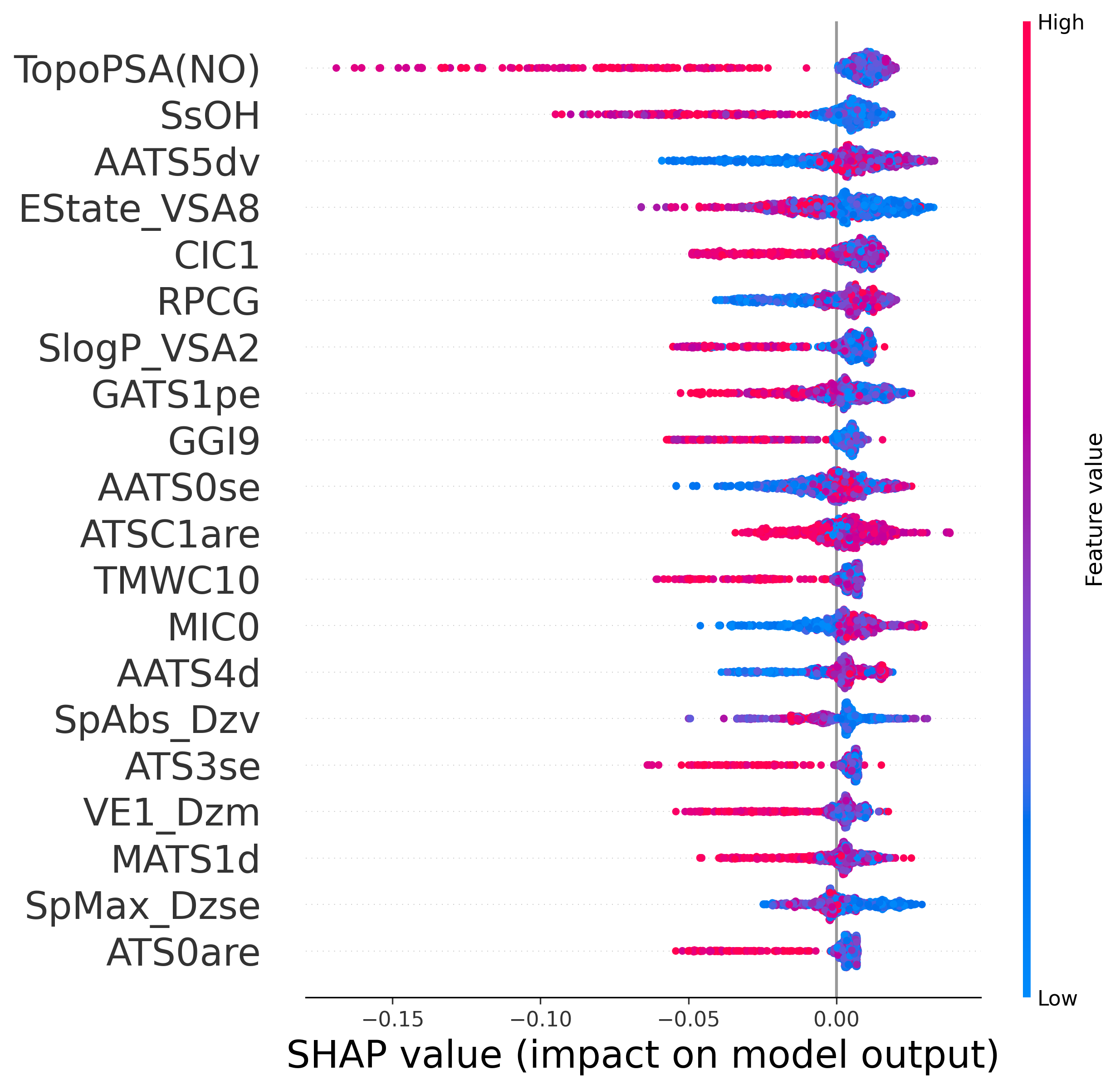
 **Figure S17**. SHAP dependence plot of the top 20 features of the RF model 2 when the cutoff is 20%.


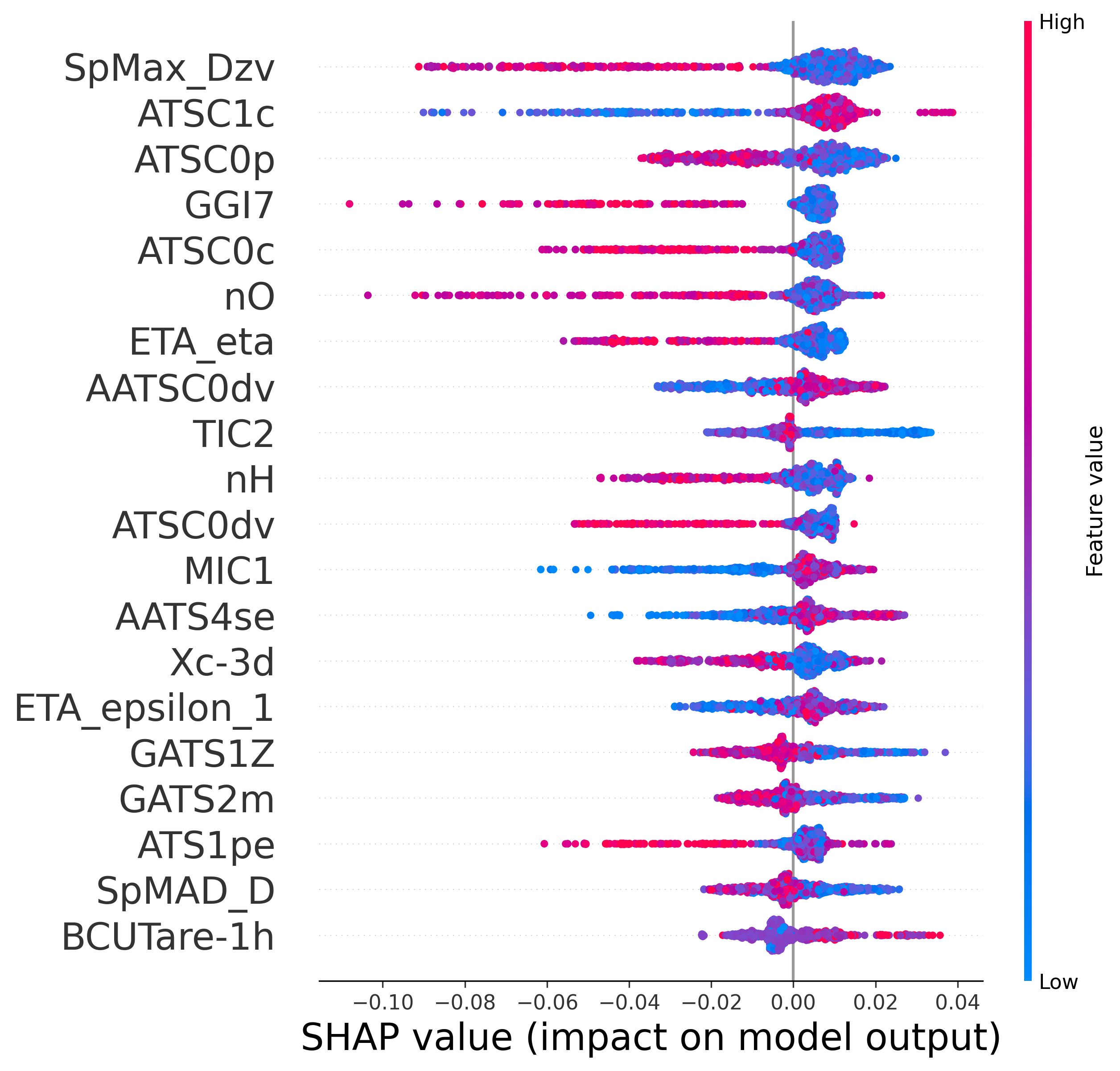
 **Figure S18**. SHAP dependence plot of the top 20 features of the RF model 3 when the cutoff is 20%.


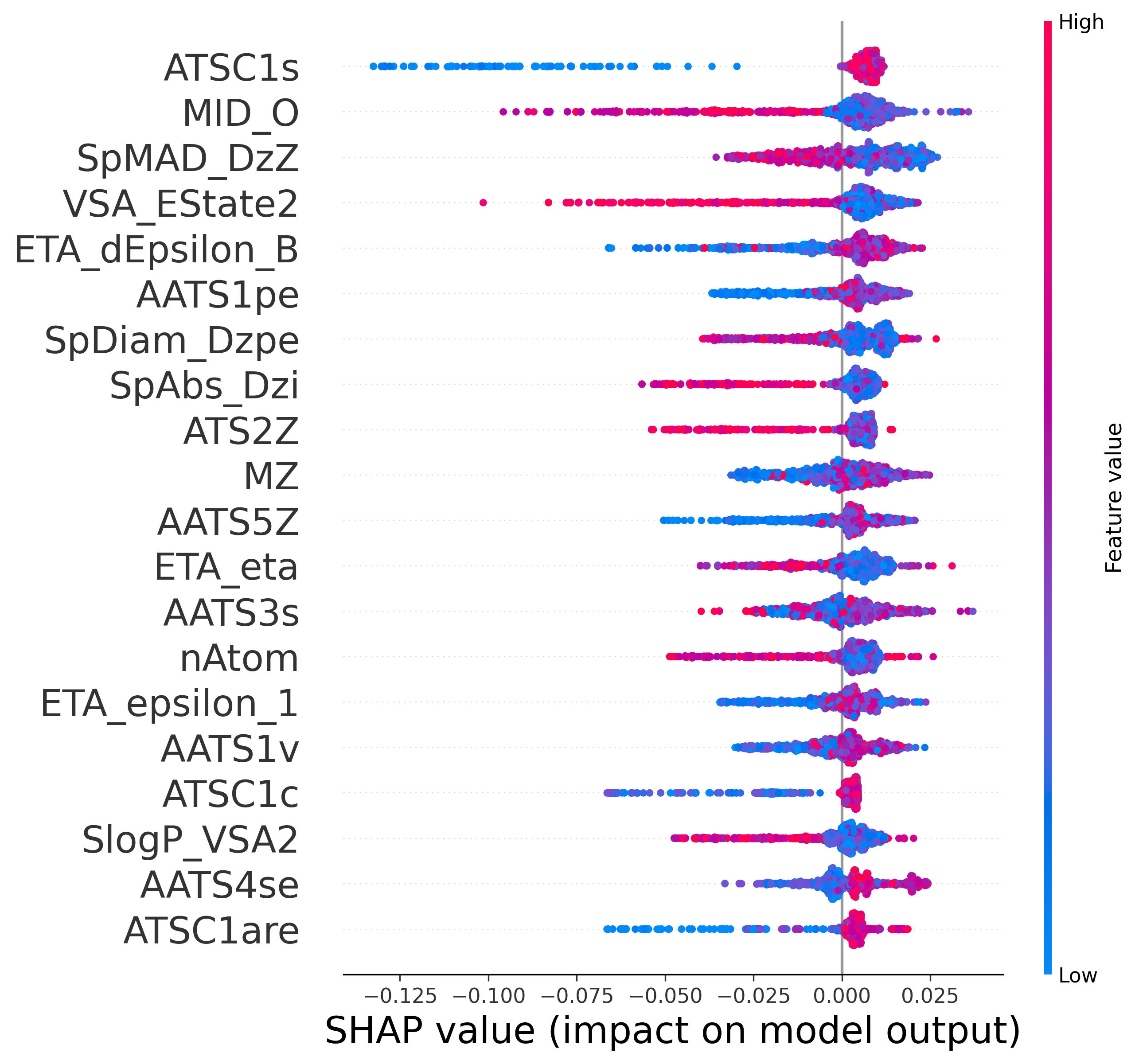
 **Figure S19**. SHAP dependence plot of the top 20 features of the RF model 4 when the cutoff is 20%.


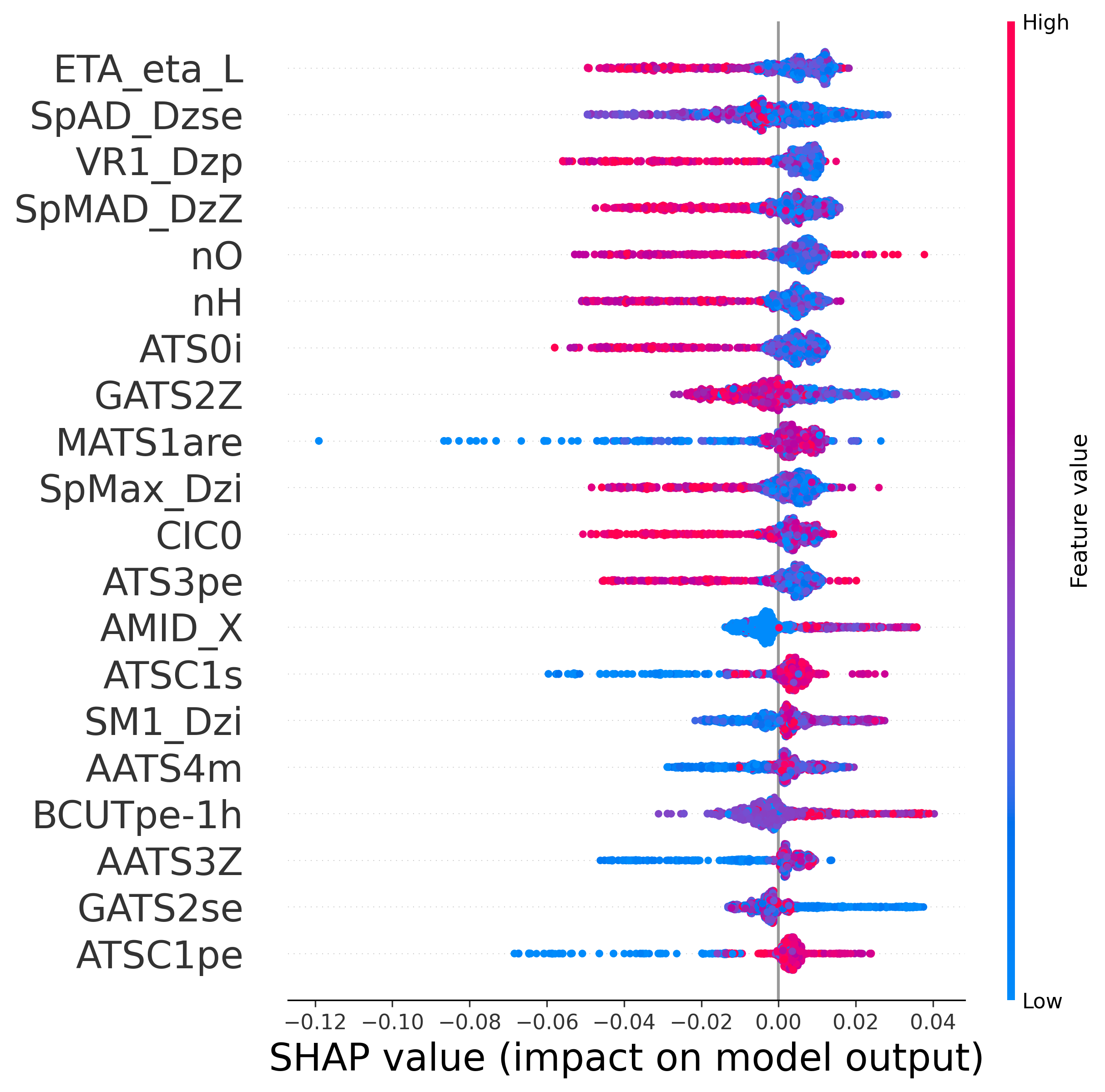
 **Figure S20**. SHAP dependence plot of the top 20 features of the RF model 5 when the cutoff is 20%.


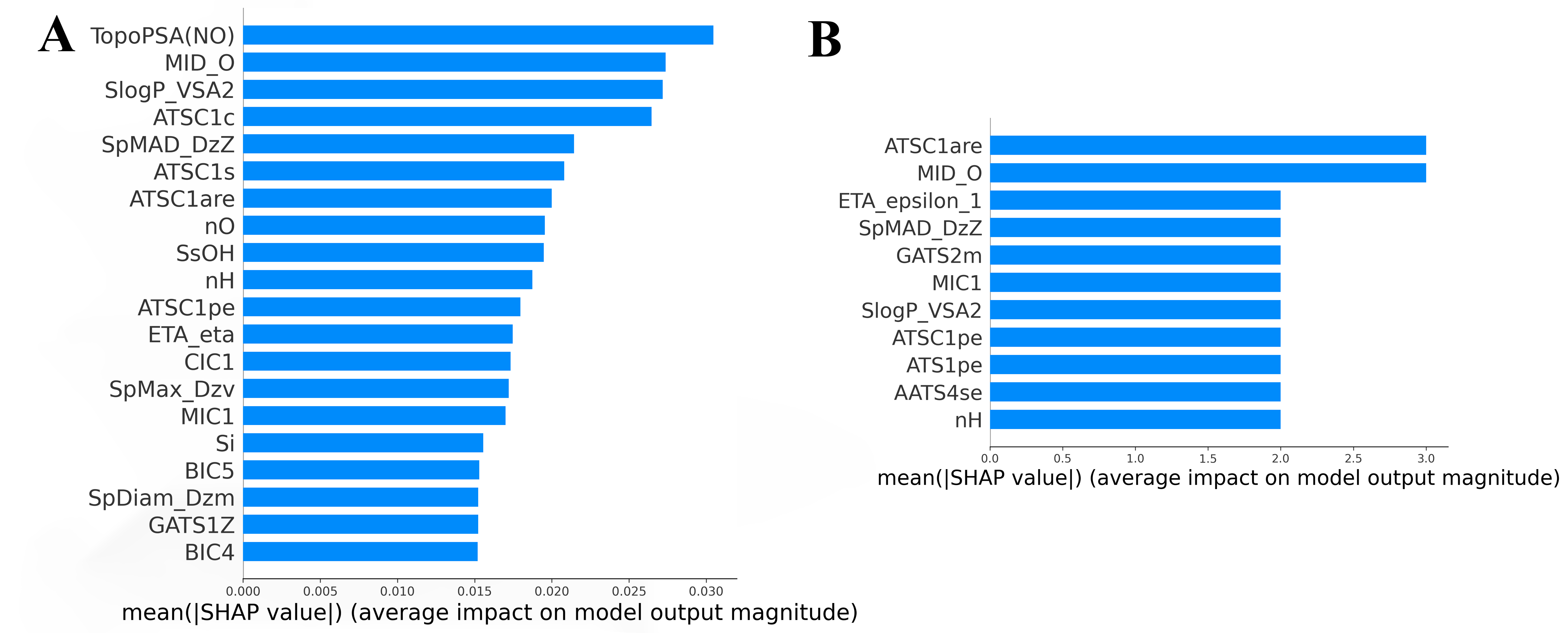
**Figure S21**. (A) Importance matrix plot of the consensus model when the cutoff is 20%. (B) A statistical graph of the number of occurrences of the top 20 features that affect all models when the cutoff is 20%


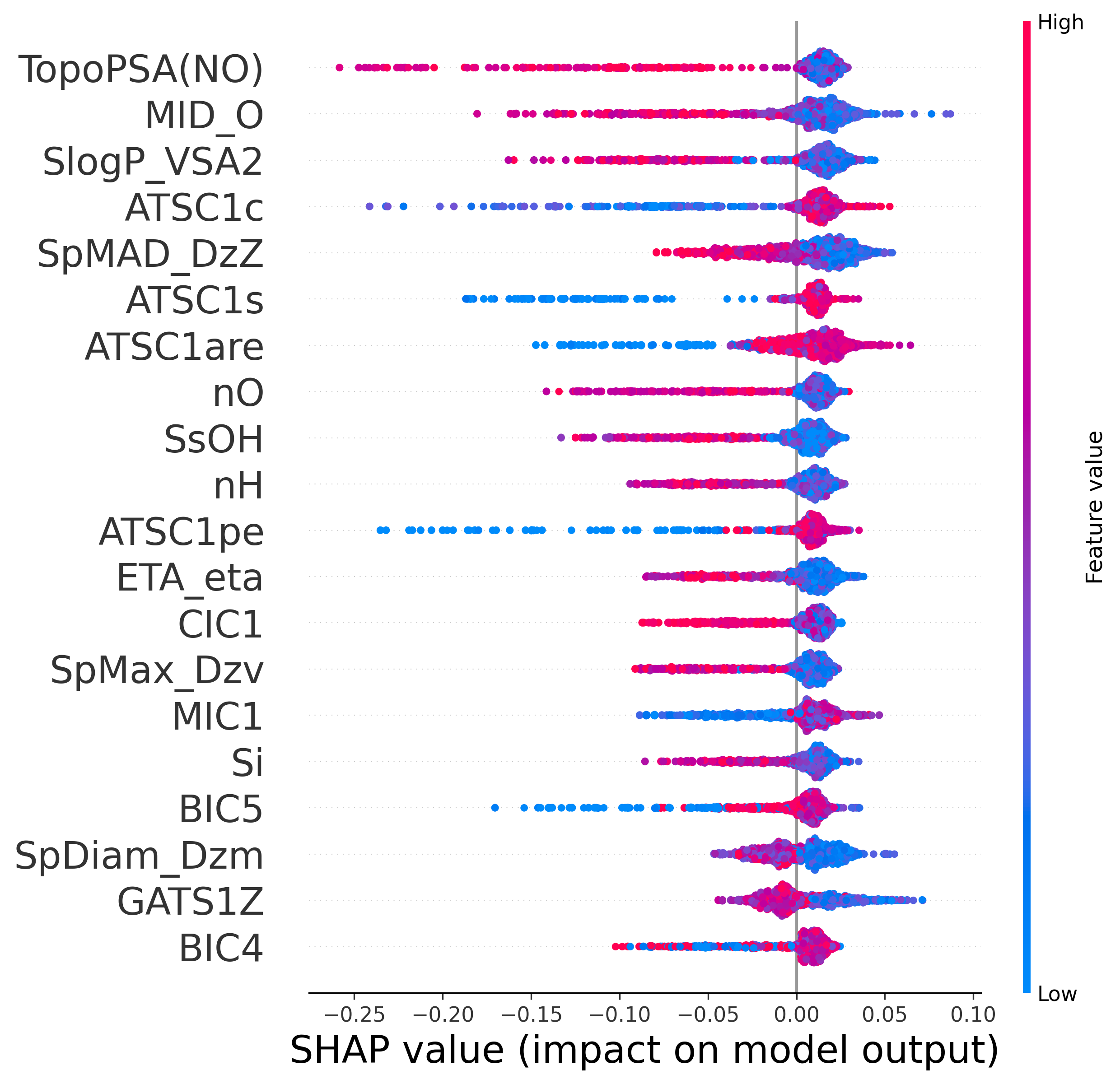


**Figure S22.** SHAP dependence plot of the top 20 features of the consensus model when the cutoff is 20%.
